# Phylogeography and morphological evolution of *Pseudechiniscus* (Heterotardigrada: Echiniscidae)

**DOI:** 10.1038/s41598-021-84910-6

**Published:** 2021-04-07

**Authors:** Piotr Gąsiorek, Katarzyna Vončina, Krzysztof Zając, Łukasz Michalczyk

**Affiliations:** Department of Invertebrate Evolution, Institute of Zoology and Biomedical Research, Faculty of Biology, Jagiellonian University, Gronostajowa 9, 30-387 Kraków, Poland

**Keywords:** Zoology, Phylogenetics, Taxonomy, Biogeography

## Abstract

Tardigrades constitute a micrometazoan phylum usually considered as taxonomically challenging and therefore difficult for biogeographic analyses. The genus *Pseudechiniscus*, the second most speciose member of the family Echiniscidae, is commonly regarded as a particularly difficult taxon for studying due to its rarity and homogenous sculpturing of the dorsal plates. Recently, wide geographic ranges for some representatives of this genus and a new hypothesis on the subgeneric classification have been suggested. In order to test these hypotheses, we sequenced 65 *Pseudechiniscus* populations extracted from samples collected in 19 countries distributed on 5 continents, representing the Neotropical, Afrotropical, Holarctic, and Oriental realms. The deep subdivision of the genus into the cosmopolitan *suillus-facettalis* clade and the mostly tropical-Gondwanan *novaezeelandiae* clade is demonstrated. *Meridioniscus* subgen. nov. is erected to accommodate the species belonging to the *novaezeelandiae* lineage characterised by dactyloid cephalic papillae that are typical for the great majority of echiniscids (in contrast to pseudohemispherical papillae in the *suillus-facettalis* clade, corresponding to the subgenus *Pseudechiniscus*). Moreover, the evolution of morphological traits (*striae* between dorsal pillars, projections on the pseudosegmental plate IV’, ventral sculpturing pattern) crucial in the *Pseudechiniscus* taxonomy is reconstructed. Furthermore, broad distributions are emphasised as characteristic of some taxa. Finally, the Malay Archipelago and Indochina are argued to be the place of origin and extensive radiation of *Pseudechiniscus*.

## Introduction

Tardigrades represent a group of miniaturised panarthropods^1^, which is recognised particularly for their abilities to enter cryptobiosis when facing difficult or even extreme environmental conditions^2^. Their relationships with Arthropoda and Onychophora are a subject of long-standing debate^3^, although the sister position with respect to these two lineages is the most popular hypothesis^4^. Internal tardigrade relationships are enigmatic likewise, despite the fact that the effort to decipher their phylogeny has been on an increase in the last decade^5–8^. The insufficient understanding of evolutionary relationships between tardigrade species (Darwinian shortfall) is not the only gap in our knowledge^9^, as it results from the scarcity of described tardigrade species^10^ (Linnean shortfall). The other parallel problem is that tardigrade distribution on the globe is vastly unknown^11^ (Wallacean shortfall), and many old records are actual misidentifications caused by poor understanding of how to dissect intraspecific and interspecific variability^12–14^. Currently, tardigradologists aim at unravelling biodiversity patterns within the phylum with the application of modern molecular tools, i.a.^15–17^.

**Figure 1:**
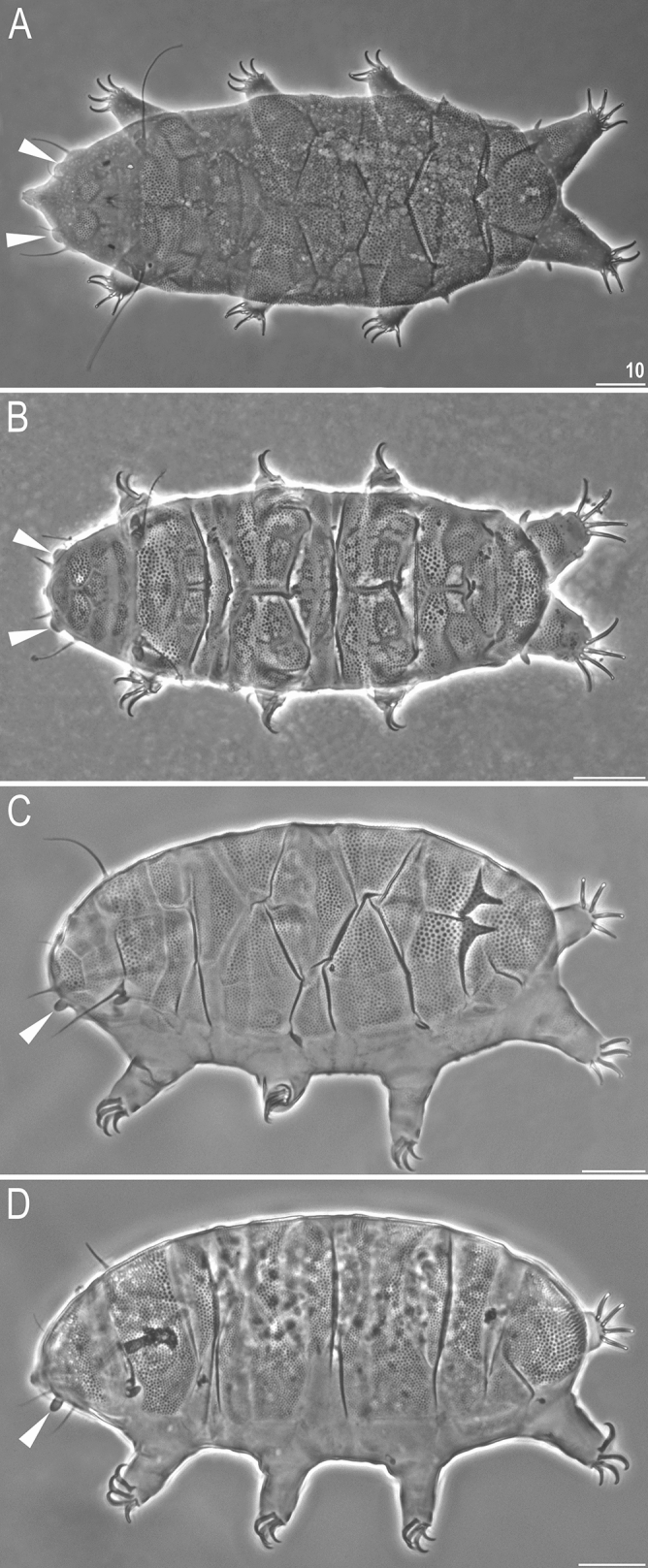
Exemplary representatives of the former *suillus-facettalis* lineage: (**A**) *Pseudechiniscus (P.) insolitus* (paratype, Spain), (**B**) *Pseudechiniscus (P.) jubatus* (paratype, Russia), and the *novaezeelandiae* lineage: (**C**) *Pseudechiniscus (M.) novaezeelandiae* (Te Ika-a-Māui), (D) *Pseudechiniscus (M.) juanitae* (Colombia). Arrowheads indicate cephalic papillae. Scale bars = 10 μm.

One of the most taxonomically absorbing conundrums of recent years concerns the genus *Pseudechiniscus*^18^, the echiniscid genus extremely scarce in morphologically informative traits^19^ after the erection of *Acanthechiniscus* to accommodate the distinct evolutionary history and morphology of some species earlier attributed to *Pseudechiniscus*^20^. The recent disclosure of the molecular diversity within *Pseudechiniscus*^21^, with an overview of morphological criteria^21–24^ and an integrative re-description of the nominal species *Pseudechiniscus suillus*^23^ constituted the turning point in the classification of this taxon. Perhaps the two most significant findings of these studies were the discovery of the crucial taxonomic role of ventral sculpturing patterns^22–24^, independently shown also for *Hypechiniscus*^25^, and the distinguishing of two phylogenetic lineages concordant with the shape of the cephalic papillae (secondary clavae)^21^, which is a unique state in the entire family, as other echiniscid genera are homogeneous in this criterion^19^. The *suillus-facettalis* lineage exhibits pseudohemispherical (illusively dome-shaped) papillae (e.g. *P. insolitus* or *P. jubatus*, Fig. 1A–B), whereas members of the *novaezeelandiae* line exhibit dactyloid papillae (e.g. *P. novaezeelandiae* or *P. juanitae*, Fig. 1C–D). Cesari et al.^21^ implied that these lines correspond with potential subgenera, simultaneously noting that the genetic evidence was insufficient at the time to erect them. From the biogeographic point of view, *Pseudechiniscus* can be superficially considered as impractical due to extreme morphological stasis^26^ that hampers correct identification of species and impedes the delimitation of geographic ranges.

In this study, we sequenced 65 populations of *Pseudechiniscus* obtained from around the world, although with over 70% of the dataset originated from the Palaearctic and Indomalayan regions. Three nuclear markers (18S rRNA, 28S rRNA, ITS-1) were used for the phylogeny reconstruction and species delimitation. Phylogenies were later exploited in a number of analyses, including biogeographic methods: S-DIVA^27^ and BioGeoBEARS^28^, ancestral state reconstruction^29^ of pivotal taxonomic traits; ecological niche modelling^30^ for some widely distributed species, and global species richness estimation in *Pseudechiniscus*. Thanks to these analyses, we present new hypotheses on the origin of the genus, stress the importance of the tropical zone (especially Southeast Asia) in the diversification not only for *Pseudechiniscus*, but tardigrades in general. We also formulate new research topics that appear as important directions in studying their diversity.

## Results

### Phylogeny and taxonomy

**Figure 2:**
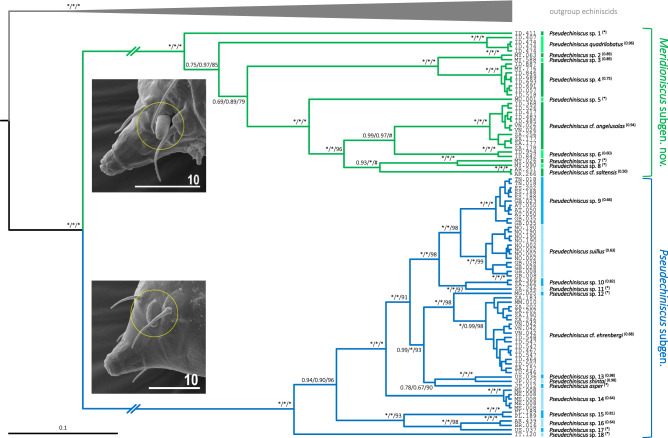
Phylogeny of the genus *Pseudechiniscus* based on the concatenated matrix (18S rRNA+28S rRNA+ITS-1). Values at nodes separated by forward slashes signify BEAST Bayesian posterior probability (BI), MrBayes Bayesian posterior probability, and bootstrap values (ML), respectively. Maximum supports, i.e. 1.00 for BI and 100 for ML, are indicated by asterisks. Support values for tip nodes (within intra-specific phylogenetic structure) are not shown for simplicity. Topologies of all consensus trees were identical, with the exception of a single clade, in which ML gave incongruent results (marked with hashtags). The scale refers to the BEAST consensus tree. Values in round brackets and in the superscript at each species name refer to the support they received in the bPTP delimitation method (maximum values, i.e. 1.00, are indicated by asterisks). Exemplary SEM microphotographs show the dactyloid papillae in *Meridioniscus* **subgen. nov.** (*Pseudechiniscus (M.)* cf. *angelusalas*) and pseudohemispherical papillae in the subgenus *Pseudechiniscus* (*Pseudechiniscus (P.)* cf. *ehrenbergi*). Scale bars in the photos in μm.

Overall, both Bayesian and Maximum Likelihood approaches gave well-resolved and largely congruent phylogenies (Fig. 2). The monophyly of *Pseudechiniscus* received a maximal support, and the genus was clearly divided in two large, deeply divergent clades, coherent with the *suillus-facettalis* (Fig. 1A–B) and *novaezeelandiae* morphotypes (Fig. 1C–D); see identical division in the COI phylogeny (Supplementary Material 2). Notably, branches in the latter are visibly longer than in the *suillus-facettalis* line. Moreover, the only inconsistent topology occurred in the *novaezeelandiae* line, in which ML recovered the following arrangement of species: ((((*P.* cf. *angelusalas* (((*P*. sp. 6 ((*P.* cf. *saltensis* (*P*. sp. 7 + *P*. sp. 8)))), but with weak support. On the basis of ITS-1 tree, the number of species estimated by bPTP varied between 23 and 43, with the average of 30. Both ML and the simple heuristic search indicated the existence of 26 species (Fig. 2), which was congruent with classical taxonomic identification. The only exception was *P.* cf. *saltensis*, divided into two putative species in the Bayesian implementation of the Poisson Tree Processes (bPTP), although with weak support (0.50). However, we classified populations AR.251 and 266 as morphologically homogeneous and largely corresponding with the original description^31^. Consequently, the number of *Pseudechiniscus* species utilised in our phylogenetic reconstruction was determined as 25.

Given that the phylogeny was compatible with morphological variation, we herein divide *Pseudechiniscus* in two subgenera: the nominal *Pseudechiniscus* (corresponding to the *suillus-facettalis* group) and *Meridioniscus* **subgen. nov.** (the *novaezeelandiae* group).

Subgenus: *Pseudechiniscus* Thulin, 1911^18^.

Type species: *Pseudechiniscus suillus*^32^.

Composition: *P. alberti*, *P. asper*, *P. beasleyi*, *P. bidenticulatus*, *P. brevimontanus*, *P. chengi*, *P. clavatus* **sp. dub.**, *P. ehrenbergi*, *P. facettalis*, *P. insolitus*, *P. jubatus*, *P. lacyformis*, *P. lalitae*, *P. megacephalus* **sp. dub.**, *P. nataliae*, *P. occultus*, *P. papillosus*, *P. pseudoconifer*, *P. ramazzottii*, *P. scorteccii*, *P. shintai*, *P. suillus*, *P. xiai*.

Diagnosis: Small echiniscids with black crystalline eyes. Only cephalic cirri present. Cephalic papillae pseudohemispherical (laterally attached to the head surface). Pseudosegmental plate IV’ present. Dorsal sculpturing of the *Pseudechiniscus* type, i.e. consisting of endocuticular pillars; usually lacking pores. *Striae* usually absent. Ventral plates absent.

Remarks: Tumanov^22^ indicated dubious character of *P. clavatus*. Not only the shape of both primary and secondary clavae is atypical, but also the pseudosegmental plate IV’ is unidentifiable in the original description based on juvenile specimens^33^. Therefore, we ascertain that *P. clavatus* is a ***nomen dubium*** and this name should not be used in modern literature. We are in agreement with Tumanov regarding the status of *P. jiroveci* **sp. dub.** and *P. marinae*. Contrarily to Grobys et al.^23^, we are of the opinion that *P. megacephalus* does not belong to a different genus. The alleged mushroom-shaped cephalic papillae with a peduncle-like bases are unknown in any other echiniscid species^19^, making this trait most likely artefactual rather than autapomorphic^34^. Consequently, *P. megacephalus* is designated as a ***nomen dubium***. *Pseudechiniscus pulcher* should be included within *Antechiniscus*^22^. *P. transsylvanicus* has long cirri *C* and the body ca. 350 μm long^35^, that is much larger than the typical length for *Pseudechiniscus*, which can reach up to 250 μm, but usually is below 200 μm. Moreover, cirriform trunk appendages are absent in *Pseudechiniscus s.s.*^22^, thus *P. transsylvanicus* almost certainly belongs in a different echiniscid genus, but the currently available data preclude its transfer. The same is for *P. bispinosus* characterised by two rigid, long spines *C*^36^. *Pseudechiniscus shilinensis* has an inadequate description preventing its taxonomic identification^37^, and is designated here as a ***nomen dubium***. Finally, the following species are unassignable to subgenera as their descriptions do not specify the cephalic papillae shape: *P. dicrani* and *P. marinae*^38,39^, thus they are tentatively retained in the subgenus *Pseudechiniscus* until type or fresh material is available.

Subgenus: *Meridioniscus* **subgen. nov.** Gąsiorek, Vončina & Michalczyk.

Type species: *Pseudechiniscus novaezeelandiae*^40^.

Composition: *P. angelusalas*, *P. bartkei*, *P. conifer*, *P. dastychi*, *P. indistinctus*, *P. juanitae*, *P. novaezeelandiae*, *P. quadrilobatus*, *P. saltensis*, *P. santomensis*, *P. spinerectus*, *P. titianae*, *P. yunnanensis*.

Diagnosis: As for the nominal subgenus, except the cephalic papillae which are dactyloid, and attached to the body cuticle only at their bases. *Striae* present, usually large and well-developed.

Etymology: From Latin *meridionalis* = southern + ending -*iscus* derived from *Echiniscus*, the first established echiniscid genus, literally meaning “an echiniscid from the South”. The name refers to the geographic origin of the known species belonging to the new subgenus, that most likely has its roots in the Southern Hemisphere and tropical/subtropical regions of the world.

Remarks: *Pseudechiniscus angelusalas*, *P. dastychi* and *P. indistinctus* were incorrectly assigned to the *suillus-facettalis* line by Roszkowska et al.^24,41^. The original description of *P*. *yunnanensis* does not specify the shape of cephalic papillae^42^, but the development of evident *striae* clearly indicates its affinity within *Meridioniscus* **subgen. nov.**

**Figure 3:**
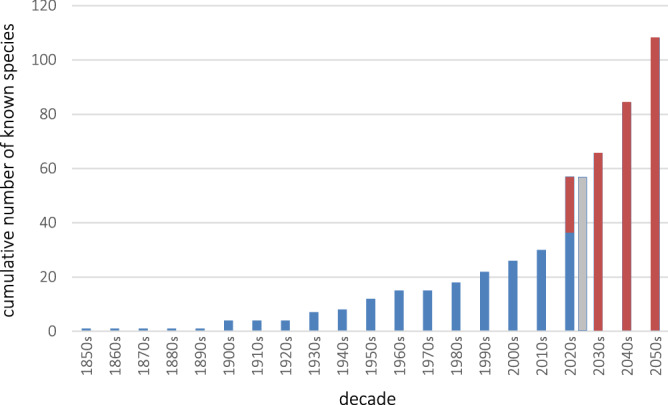
Cumulative species richness of *Pseudechiniscus* since the description of the nominal *Pseudechiniscus suillus* with predicted species numbers until 2059. Red bars indicate the predicted number of species in the future decades. The decade 2020–2029 is the only one with two bars: the first bar signifies the number of described species (blue portion) + the predicted number of species (red portion), whereas the second (grey) bar signifies the total number of known and undescribed species that were uncovered in this study. See sections Remarks in the Results for the taxonomy and our additional remarks on the validity of some species, echoing Tumanov^22^ and Grobys et al.^23^ findings.

The estimation of the number of *Pseudechiniscus* species to be described by the year 2050 was performed using the exponential model, by applying the best-fit curve to data on cumulative species richness. Curve was described by the formula y = 0.5791e^0.2491x^, where x signified the subsequent decades since the description of *P. (P.) suillus* in 1853 until now. The diagram showing the predicted increment in the number of *Pseudechiniscus* species is shown in Fig. 3.

### Morphological evolution and the evolution of reproductive mode

**Figure 4:**
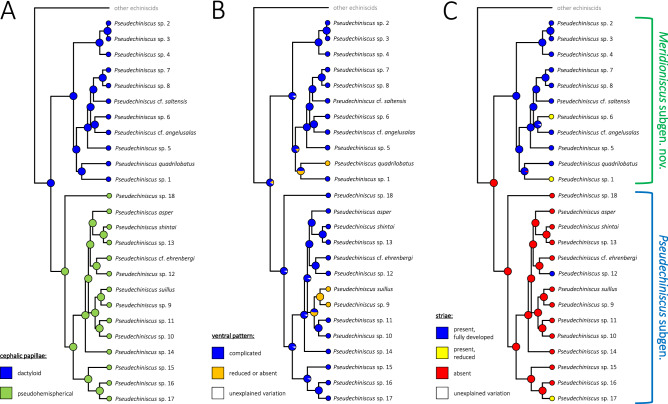
Ancestral state reconstruction and trait evolution in *Pseudechiniscus*: (**A**) the shape of cephalic papillae, (**B**) the type of ventral ornamentation, (**C**) the development of *striae* between endocuticular pillars. Pie charts illustrate the probability that a given character state occurred at a node.

**Figure 5:**
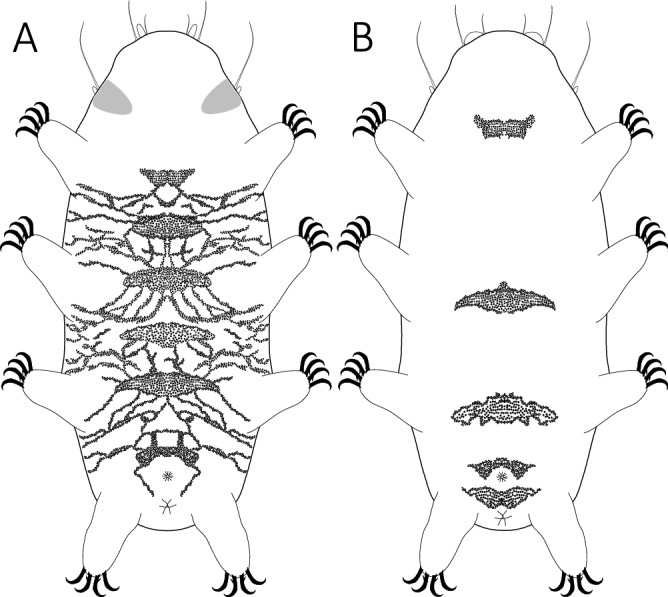
Two general ventral ornamentation patterns found within *Pseudechiniscus*: (**A**) complex (*Pseudechiniscus (M).* cf. *angelusalas*), (**B**) reduced (*Pseudechiniscus (P.)* sp. 9).

Firstly, we assessed the phylogenetic signal using 1000 most credible BEAST trees, taking four key taxonomic traits and reproductive mode into consideration: (1) the shape of cephalic papillae, (2) the type of ventral ornamentation pattern, (3) the development of projections on the posterior margin of the pseudosegmental plate IV’, (4) the development of *striae* and (5) the presence of males in a population. Of these, *p*-value for Pagel’s λ was >  > 0.05 for traits 3. and 5., thus they were discarded from further analyses. Subsequently, BayesTraits implemented in RASP was used to reconstruct ancestral states along phylogenetic lineages of *Pseudechiniscus*. The analysis showed that the ancestor of *Pseudechiniscus* had elongated (dactyloid) cephalic papillae, therefore the pseudohemispherical papillae of the nominal subgenus should be considered autapomorphic (Fig. 4A; Pagel’s λ_1_ = 1.00, *p* < 0.001). Furthermore, ancestors of *Pseudechiniscus* and its subgenera were most likely characterised by complex ventral ornamentation patterns (Figs. 4B, 5A; Pagel’s λ_2_ = 1.00, *p* < 0.001), but parallel simplification of the ventral sculpturing (Fig. 5B) occurred at least twice in the course of evolution: (I) in the tropical lineage comprising *P. (M.) quadrilobatus* and *P. (M.)* sp. 1, and (II) in the Palaearctic lineage represented by *P*. *(P.)* sp. 9 and *P. (P.) suillus*. Finally, *striae* were shown as absent in ancestors of *Pseudechiniscus* and its nominal subgenus, being a derived and a conservative trait in *Meridioniscus* **subgen. nov.** (Figs. 4C; Pagel’s λ_4_ = 0.94, *p* < 0.001). *Striae* did, however, evolve independently also in two lineages of the subgenus *Pseudechiniscus*: *P*. *(P.)* sp. 12 and *P*. *(P.)* sp. 17 (fully developed and incipient, respectively), and they are partially reduced in two species of *Meridioniscus* **subgen. nov.** (*P*. *(M.)* sp. 1 and *P*. *(M.)* sp. 6).

### Biogeography

**Figure 6:**
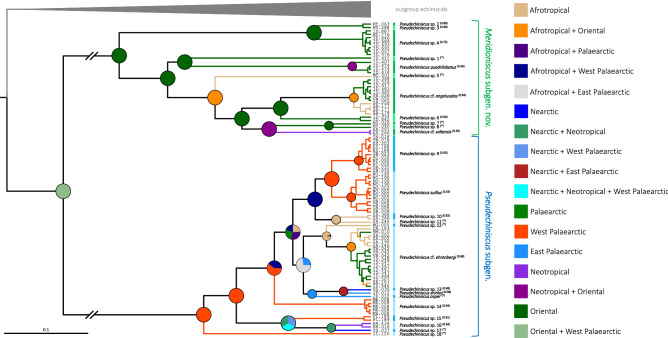
Historical biogeography of *Pseudechiniscus* reconstructed using S-DIVA. Nodal pie charts show relative probabilities of historical geographic ranges according to the in-figure colour legend. Black colour signifies unexplained origin.

**Figure 7:**
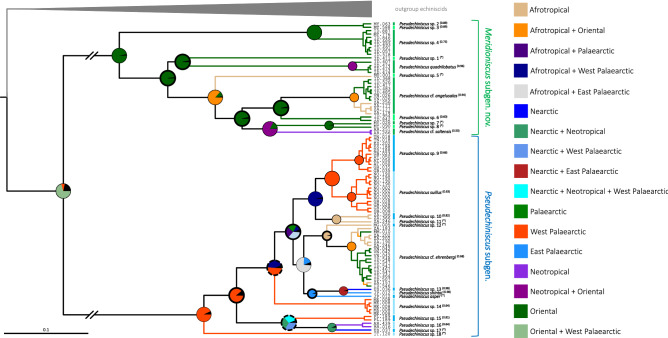
Historical biogeography of *Pseudechiniscus* reconstructed using BioGeoBEARS under the DIVALIKE model. Nodal pie charts show the relative probability of historical geographic ranges according to the in-figure colour legend. Pie charts with bolded margins denote points of dispersal, whereas these with discontinuous margins indicate a vicariant origin. Black colour signifies unexplained origin.

**Figure 8:**
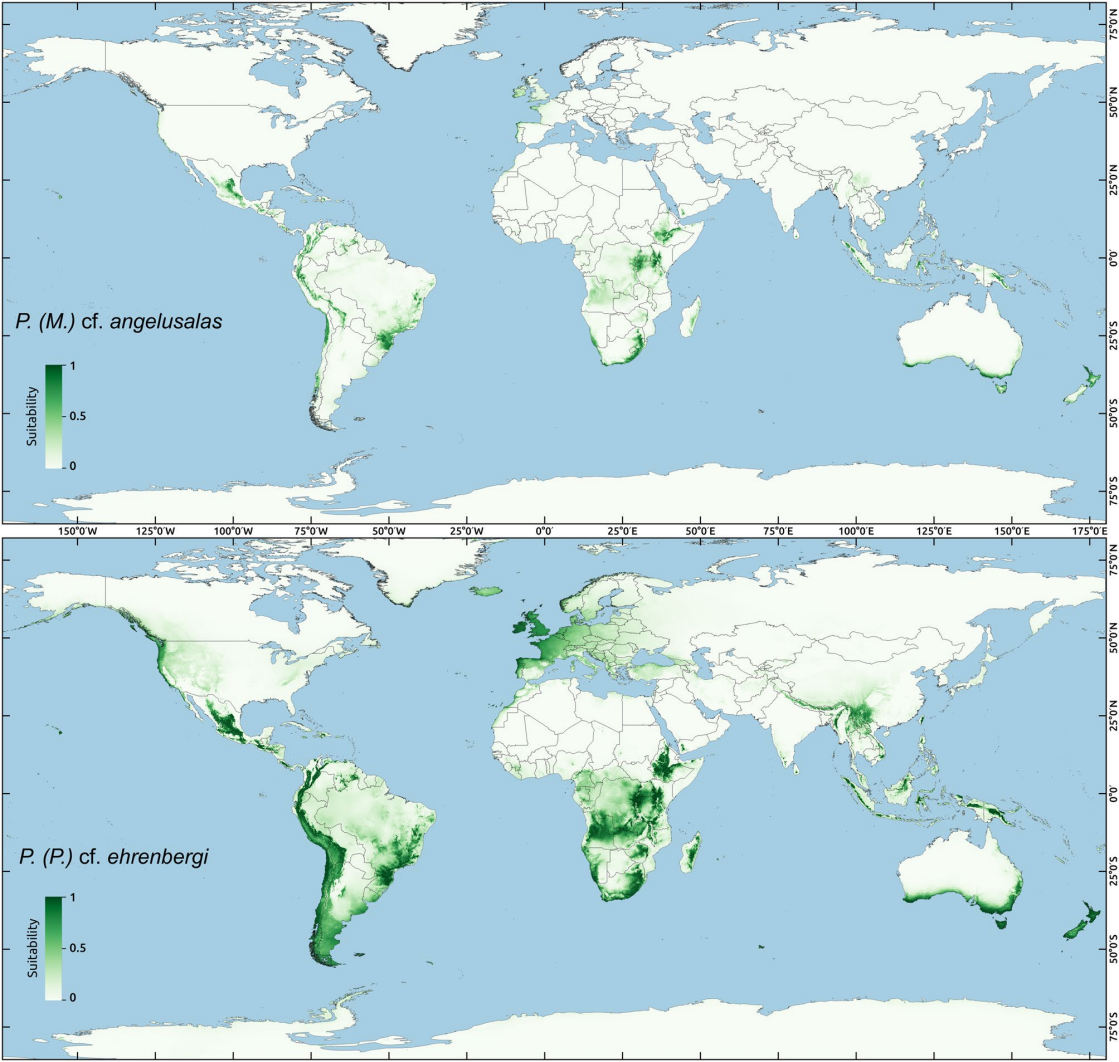
Ecological biogeography of two *Pseudechiniscus* species with pantropical distributions – geographic ranges predicted by ecological niche modelling for: (**A**) *P. (M.)* cf. *angelusalas*, (**B**) *P*. *(P.)* cf. *ehrenbergi*. Suitability determines whether a given area is characterised by favourable conditions for one of the species (maximal suitability = 1) or by allegedly inhospitable conditions (minimal suitability = 0). Generated using Maxent, ver. 3.4.1^103^ [https://biodiversityinformatics.amnh.org/open_source/maxent/] with kuenm package^104^.

S-DIVA and ancestral range reconstruction under the DIVALIKE model gave similar results (Figs. 6–7) which suggested that the ancestor of *Pseudechiniscus* was of the West Palaearctic-Oriental origin, which is not a conclusive inference (see Discussion for the issue of geographic sampling bias, especially the lack of data for Australian lineages). However, the ancestors of the two subgenera were clearly identified as originating from the West Palaearctic (*Pseudechiniscus*) and Oriental/Indomalayan region (*Meridioniscus* **subgen. nov.**). Although the majority of lineages within *Meridioniscus* **subgen. nov.** share the Oriental origin, the picture is more complex in the subgenus *Pseudechiniscus*, as the basal lineages originated in the West Palaearctic, but the remaining ones have a composite character (not discussed in detail since a broader sampling within *Pseudechiniscus* is likely to give disparate results in the deep nodes within the subgenera compared to our study). Moreover, two species have wide tropical distributions: *P. (M.)* cf. *angelusalas*, with pronounced diphyletic structure (Afrotropical and Oriental lineages), and *P*. *(P.)* cf. *ehrenbergi*, with two clades of mixed origin (Figs. 6–7). The analysis under the DIVALIKE model revealed two points of vicariance in *Meridioniscus* **subgen. nov.**, and six analogous points in *Pseudechiniscus*. Additionally, there were four points of dispersal in *Meridioniscus* **subgen. nov.** (from the Oriental region or within this area), and six analogous points in the subgenus *Pseudechiniscus* (between many regions). The modelling of potential distributions based on available incidence data was performed for two species with molecularly verified records from various realms: *P*. (*M*.) cf. *angelusalas* and *P*. (*P*.) cf. *ehrenbergi* (Fig. 8). Whereas the distribution of *P*. (*M*.) cf. *angelusalas* was inferred as almost strictly subtropical-pantropical, the modelled distribution of *P*. (*P*.) cf. *ehrenbergi* extended towards temperate regions in the Americas (Cascade Range and Patagonia) and in Western Europe (Fig. 8).

## Discussion

Systematic and morphological studies with the application of phylogenetic and statistical methods on meiofaunal phyla, represented e.g. by tardigrades^43^, are notoriously laborious and challenging. The finding of abundant populations of *Pseudechiniscus* allowed for the confirmation of hypotheses^21,22^ that its two evolutionary lineages correspond with some morphological traits and can be concurrently elevated to the rank of subgenera. Among the examined traits, the shape of cephalic papillae was recovered as the most conservative character, followed by the development of *striae*, and the most labile traits, namely the type of ventral ornamentation (Fig. 4) and the development of projections on the posterior margin of the pseudosegmental plate IV’. Pseudohemispherical cephalic papillae occur only in the subgenus *Pseudechiniscus*, but their hemispherical appearance under LCM makes them superficially similar to truly hemispherical papillae in *Parechiniscus*^19^, *Cornechiniscus*^19,44^, *Mopsechiniscus*^45^ or *Proechiniscus*^19^. Moreover, the cephalic papillae in *Novechiniscus*, being very wide and with additional cuticular ring at the base, but at the same time pointed at their tips^46^, are intermediate in shape between the spherical and dactyloid types. All other echiniscid genera have elongated papillae, present also in Oreellidae^47^. However, Carphaniidae do not have papillae^47,48^, and Echiniscoididae have strongly modified papillae that are comparatively large with respect to the size of the head and are hemispherical in shape^49–51^. Not excluding the homologous character of the shape of the cephalic papillae in some Echiniscidae and Echiniscoididae, we are inclined to hypothesise that hemispherical and pseudohemispherical papillae are derived states, present only in some lineages of the clade Echiniscoidea.

As noted by Tumanov^22^, the presence of *striae* in the dorsal sculpturing correlates with phylogeny. *Striae* are a derived state present in *Meridioniscus* **subgen. nov.** and only rarely occur in the subgenus *Pseudechiniscus* (Fig. 4C). However, some of the examined species, for which multipopulation data are available, exhibit intraspecific variability in the development of *striae* (e.g. *P*. *(P.)* sp. 9 and *P. (M.)* cf. *angelusalas*, see Supplementary Material 2). Their homoplasious convergent nature is supported by the independent development in several not directly related lineages within Echiniscidae, e.g. in *Cornechiniscus*^44^, *Stellariscus*^52^, or some *Echiniscus* species, such as *E. rackae*^53^. Importantly, the ancestor of the genus *Pseudechiniscus* did not exhibit *striae*, thus its dorsal sculpturing probably resembled that in *Hypechiniscus*^25^. Such a type of sculpturing, composed solely of endocuticular pillars, is plesiomorphic for Echiniscidae in general. Consequently, we recognise the development of pores or a sponge layer as advanced characters that evolved convergently in the *Bryodelphax* and *Echiniscus* lineages^44^.

Ventral ornamentation pattern only recently was found to be pivotal in the taxonomy of *Pseudechiniscus*^22–24,41^, albeit a reticulated design was reported in many species long before these analyses^54^. Although taxonomically informative, this trait is not easily identifiable and requires an optical equipment of high quality and properly fixed specimens to comprehensively describe the belts of pillars and connective points. Moreover, inter-population variability was observed in some widely distributed species, such as *Pseudechiniscus (M.)* cf. *angelusalas*, *P*. *(M.)* sp. 4 or *P*. *(P.)* cf. *ehrenbergi*, evidenced particularly in the intensity of sculpturing in the intersegmental areas between the subsequent pairs of limbs. Moreover, our populations of *P. (P.) suillus* exhibited reduced ventral sculpturing, whereas the redescription provides an intricate pattern in this species^23^. The absence of ventral ornamentation is a derived character in the genus *Pseudechiniscus*, so far observed only in *P. (M.) quadrilobatus* (replaced by a system of epicuticular thickenings known in *Cornechiniscus*^44^). This species has strongly sclerotised armour, and the examination of the type material of *P. alberti*, also having unusually for the genus sclerotised plates^55^, would be desirable to adjudicate whether the reduction of ventral sculpturing is correlated with the strengthening of the dorsal armour.

Projections, in the form of teeth or lobes, occur on the posterior margin of pseudosegmental plate IV’ in both *Pseudechiniscus* subgenera. The variability in the development of the projections varies between species, as some are known to have relatively invariant projections, especially taxa having large teeth, like *P. (M.) novaezeelandiae* or *P. (M.) spinerectus*^56,57^, but some other, particularly species with weakly elevated teeth or lobes, such as *P. (P.) asper* or *P. (M.) santomensis*^41,58^, are characterised by a considerable number of intermediate stages: from the fully developed projections to an almost completely smooth edge of the plate. Such variability was also observed in the newly sequenced species, such as e.g. *P*. *(M.)* sp. 6 and *P*. *(P.)* sp. 17. Another interesting finding is the presence of the clade *P. (M.) quadrilobatus* + *P*. *(M.)* sp. 1 in the ancestral state reconstruction analysis (Fig. 4), indicating spurless claws as autapomorphic. Noteworthy, the Javanese *P. (P.) bidenticulatus* also has spurless claws^59^, suggesting its affinity to this clade and a potential transfer to *Meridioniscus*. Internal claws with primary spurs are plesiomorphic to Echiniscidae, and the loss of spurs evolved several times in different genera, e.g. in *Cornechiniscus*^44^. Irrespectively of this, *Pseudechiniscus* exhibits a tendency towards miniaturisation of spurs and their further merging with the claw branch, especially in the Asian lineages^41^.

Our analyses show that *Pseudechiniscus* biogeography is complex and requires a detailed interpretation. Our initial assumption was in accordance with the ‘everything is everywhere, but environment selects’ hypothesis^60,61^, i.e. that the majority of species may have wide geographic distributions, but are limited by climate, and if exceeding a single biogeographic region^62^, then they should occur in realms with similar habitats (e.g. Oriental – Afrotropical – Neotropical). Premises fur such zero hypothesis are numerous: (1) we have already demonstrated a pantropical distribution for an echiniscid species^14^, which we expect to be the only pattern of a widespread geographic range in the tropical region (in other words, e.g. a combination Nearctic-Afrotropical should be considered as rather non-parsimonious and unlikely); (2) a wide distribution was shown for *Pseudechiniscus (P.) ehrenbergi*, which inhabits both the Western (Italy) and the Eastern (Mongolia) Palaearctic^21,24^; (3) *Meridioniscus* **subgen. nov.** was preliminarily identified in our samples from Chile, Colombia, French Guyana, Kenya, Tanzania and India (not sequenced due to the scarcity of individuals; see also an unidentified species from Argentina in Tumanov^22^), but never in a much richer material from the Holarctic (see below for the case of *P. (M.) indistinctus*); contrarily, individuals of the subgenus *Pseudechiniscus* were found worldwide, and particularly frequently in Europe, including Iceland, Germany, Sweden, Poland, Croatia, or Italy (not sequenced for identical reasons as above). These observations implied a certain degree of regionalisation at least for one of the evolutionary lineages of the genus *Pseudechiniscus*; (4) much better studied micrometazoans, such as mites, are known to be geographically regionalised, with a low proportion of pantropical and cosmopolitan species inhabiting a given region (up to only 10–15% of the entire fauna^63,64^); similar values were reported for collembolans (20%)^65^, but see the contrasting evidence for gastrotrich^66^ or proturan^67^ tropical faunae. In our dataset, out of 14 tropical *Pseudechiniscus* species, only three (*P. (P.)* cf. *ehrenbergi*, *P. (M.)* cf. *angelusalas* and likely *P. (M.) quadrilobatus*, see Supplementary Material 2) have wide geographic ranges (21%). Thus, it seems that regional endemism prevails over pantropical or cosmopolitan ranges of *Pseudechiniscus* spp. In other words, only two of the analysed species, *P. (P.)* cf. *ehrenbergi*, *P. (M.)* cf. *angelusalas*, exhibit genetically verified geographic ranges that are in agreement with the ‘everything is everywhere, but environment selects’ hypothesis (in the case of species with preferences for tropical habitats, pantropical distributions are predicted by the hypothesis).

The analyses indicated the West Palaearctic-Oriental (Indomalayan) origin of *Pseudechiniscus*, which is not surprising given the geographically biased dataset. It is probable that sequencing Antarctic (post-Gondwanan)^16^ and Australian representatives of *Pseudechiniscus* may affect the inference about the ancestral geographic range. Although *Pseudechiniscus (M.) dastychi* and *P. (M.) titianae* are autochthonous for Antarctica and there are some barcodes available for these species^20,24^, they are non-homologous with our DNA sequences, thus we were not able to include them in the reconstructions. However, it is Australia, where the crucial phyletic lineages of the genus are most likely to be found. This is so, because ancient and highly diversified lineages are known within many animal groups in Australia^68–71^. Unluckily, the Australian tardigrade fauna is almost unknown, although there are at least few *Pseudechiniscus* species representing both subgenera present on this continent^72^.

The West Palaearctic and Oriental regions were recovered as ancestral for the subgenus *Pseudechiniscus* and for *Meridioniscus* **subgen. nov.**, respectively (Figs. 6–7). Moreover, the first subgenus exhibits a cosmopolitan distribution, whereas the second is primarily tropical/subtropical-Gondwanan (although we do not preclude its presence in e.g. the Mediterranean Palaearctic). A puzzling exception to this pattern is the genetically verified presence of *P. (M.) indistinctus* in the southern part of Norway (western Scandinavian Peninsula)^24^, but the tropical origin of *Meridioniscus* **subgen. nov.** does not exclude dispersal to the Holarctic. Placing this species in the generic phylogeny will allow for the clarification of its affinities. Comparatively longer branches in *Meridioniscus* **subgen. nov.** may be caused by two factors: (1) undersampling of lineages, or (2) a relatively slower rate of speciation and extinction, accompanied by morphological stasis, suggesting bradytely (arrested evolution) in this evolutionary line^73^. This hypothesis finds support in the main retained plesiomorphy of the subgenus – dactyloid cephalic papillae (Fig. 4A).

We stress that ancestral area reconstructions using BioGeoBEARS (Fig. 7) indicated the Oriental region to be the centre of dispersal of *Meridioniscus* **subgen. nov.** lineages (four points of dispersal), which stays in agreement with the wide geographic distributions of *P. (M.) quadrilobatus* (potential dispersal to Neotropics from tropical Asia) and *P. (M.)* cf. *angelusalas* (potential dispersal to the Afrotropic from tropical Asia). Among the sequenced 25 species, only one representative of the subgenus *Pseudechiniscus* exhibited a similarly wide tropical distribution (*P*. *(P.)* cf. *ehrenbergi*). This means that only ca. 12% of species in our dataset show signs of pantropical ranges, and we anticipate this fraction to be similar in many other cosmopolitan tardigrade genera (based also on the echiniscid-rich material from South Africa, data in preparation). The analyses predicted that *P*. (*M*.) cf. *angelusalas* and *P*. (*P*.) cf. *ehrenbergi* are likely to be pantropical (which resembles the analyses conducted on *Echiniscus lineatus*^14^), with the majority of suitable areas present in the subtropical and tropical zones, and highly suitable areas in Western Europe for *P*. (*P*.) cf. *ehrenbergi*. This species most likely inhabits also the Palaearctic, as indicated by COI data, placing our sequences with those published previously^21,24^ in one clade (Supplementary Material 2). We also stress the evident importance of mountain ranges throughout the modelled geographic ranges (Fig. 8) for the presence of both taxa, which is in line with the known pattern of tardigrade diversity and endemism increasing to some extent in relation to the elevation^74^ (also observed in many other animal groups^75^).

*Pseudechiniscus* is the second most speciose echiniscid genus after *Echiniscus*^23^, and its taxonomy was for long considered disorganised and distrustful. For example, a considerable fraction of literature reports of *Pseudechiniscus* constitute the records of *P. (P.) suillus* and *P. (P.) facettalis*^11^, two allegedly cosmopolitan species. Paradoxically, it is likely that they both have restricted geographic ranges, as their *loci typici* are at high elevations in the mountains of West Palaearctic^23,32^ and in Greenland^76^, i.e. places that favour typical stenotherms rather than eurytopic species. Our model (Fig. 3) predicts that by the mid of twentieth century, the number of described *Pseudechiniscus* species will be likely at least doubled, approaching the currently known number of *Echiniscus* species. In fact, it would not be surprising if it is actually *Pseudechiniscus* that will have the status of the most speciose echiniscid, but testing this supposition requires strict taxonomic regime, that is redescriptions of “old” taxa, e.g. *P. (P.) facettalis* or *P. (M.) conifer*, joined with descriptions of new species always associated with DNA barcodes. This will help to avoid synonymies and will also allow for supplementation of generic phylogenetic tree, as well as for more detailed biogeographic analyses and testing of the hypotheses put forward in this study. Beside of the abovementioned Australia, we foresee that sampling in the Oriental region may bring further improvement to our understanding of the natural history of *Pseudechiniscus*. Recently, Borneo and Indochina, i.e. areas that arose at the border between Laurasia and Gondwana, were argued as crucial for Oriental biodiversity^77^. This goes in tandem with what we discovered for *Pseudechiniscus*, thus tardigradologists interested in taxonomy and phylogeny of the genus are likely to benefit from focusing on the Indopacific area.

## Material and methods

### Sampling, microscopy and imaging

*Pseudechiniscus* specimens were extracted from 65 samples collected throughout the world (Supplementary Table 1). Each population was divided into at least two groups destined for DNA sequencing and light microscopy analyses. If a population was sufficiently abundant, specimens were preserved also for scanning electron microscopy. Permanent slides were made from some individuals using Hoyer’s medium and later examined under an Olympus BX53 light microscope with phase contrast (PCM), associated with an Olympus DP74 digital camera. Supplementary figures illustrating dorsal/ventral sculpturing of females and males (provided always in this order) were assembled in Corel Photo-Paint X6, ver. 16.4.1.1281. For structures that could not be satisfactorily focused in a single light microscope photograph, a stack of 2–6 images was taken with an equidistance of ca. 0.2 mm and assembled manually into a single deep-focus image.

### Cephalic papillae (secondary clavae) morphotypes

**Figure 9:**
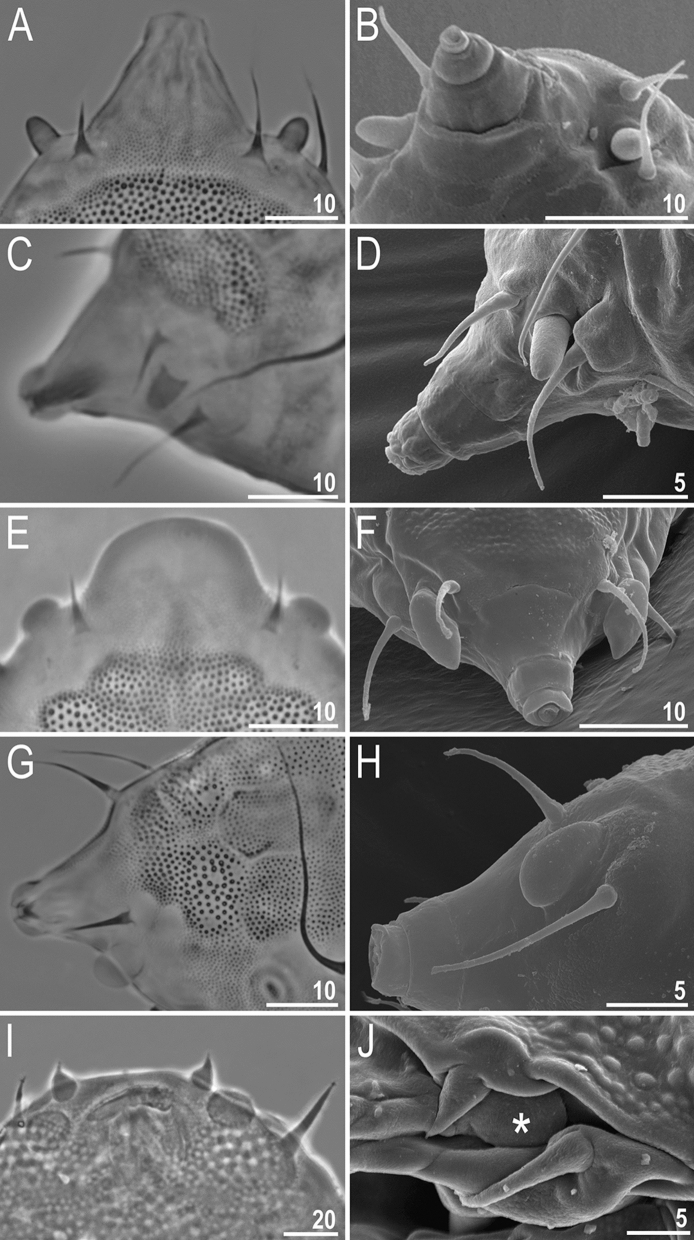
Three morphotypes of cephalic papillae in Echiniscidae: **Dactyloid papillae**, present in the subgenus *Meridioniscus* and the great majority of echiniscids, i.e. finger-like papillae attached to the body cuticle only at their bases, visible as such both under PCM and SEM: (**A**) *P. (M.)* cf. *saltensis* (AR.251, PCM, dorso-ventral projection), (**B**) *P. (M.)* sp. 5 (MU.001, SEM, ventral projection), (**C**) *P. (M.)* cf. *angelusalas* (ZA.177, PCM, lateral projection), (**D**) *P. (M.)* cf. *angelusalas* (ZA.177, SEM, lateral projection); **Pseudohemispherical papillae**, present only in the subgenus *Pseudechiniscus*, i.e. finger-like papillae attached to the body cuticle at their bases but also longitudinally, along their length, which makes them appear under PCM as hemispheres/domes, but the actual finger-like shape of the papillae is clearly visible under SEM: (**E**) *P. (P.)* cf. *ehrenbergi* (ID.546, PCM, dorso-ventral projection), (**F**) *P. (P.)* sp. 9 (TN.018, SEM, frontal projection), (**G**) *P. (P.)* sp. 9 (ES.202, PCM, dorso-lateral projection), (**H**) *P. (P.)* sp. 9 (TN.018, SEM, lateral projection); **Hemispherical papillae**, i.e. truly hemispherical/dome-shaped papillae, flattened and widened but attached to the body cuticle only at their bases, visible both under PCM and SEM (present in *Cornechiniscus*, *Mopsechiniscus*, *Parechiniscus*, *Mopsechiniscus* and *Proechiniscus*): (**I**) *Cornechiniscus madagascariensis* (paratype, PCM, ventral projection), (**J**) *Cornechiniscus madagascariensis* (ET.007, SEM, lateral projection). Figs **I**–**J** from^44^. Scale bars in μm.

Although all species in the genus *Pseudechiniscus* have elongated cephalic papillae, the subgenera *Pseudechiniscus* and *Meridioniscus* **subgen. nov.** differ in the orientation and attachment of the papillae to the cuticle of the body, which results in their very much different appearance under LCM (Fig. 9)^22^. Specifically, papillae in *Meridioniscus*, as in the great majority of Echiniscidae, are attached to the body only by their base and they appear finger-like under both SEM and LCM (Fig. 9A–D). However, in the subgenus *Pseudechiniscus*, the papillae are attached to the head also along their side (Figs. 9E–H). This peculiar longitudinal attachment is clearly visible in SEM (Fig. 9F,H), but most often makes these papillae appear in LCM as hemispherical (dome-shaped)^21^ (Fig. 9E,G). Thus, under LCM, cephalic papillae in the subgenus *Pseudechiniscus* resemble those in *Cornechiniscus* (Fig. 9I–J) and *Mopsechiniscus*. However, the papillae in the two latter genera are flattened and widened from the apex, thus they are indeed hemispherical (dome-shaped) and are visible as such in both SEM and LCM. Thus, in order to avoid confusion when describing the shape of cephalic papillae in the subgenus *Pseudechiniscus*, and to differentiate them from the truly hemispherical (dome-shaped) papillae in *Cornechiniscus* (Fig. 9I–J) and *Mopsechiniscus*^45^, we propose to term them as pseudohemispherical.

### Comparative material

We borrowed and examined the following type specimens of *Pseudechiniscus* species: *P. bartkei*, *P. gullii*, *P. insolitus*, *P. jubatus*, *P. nataliae*, *P. quadrilobatus*, *P. ramazzottii*, *P. santomensis*, *P. spinerectus*. They were used to determine their subgeneric affinity and to formulate taxonomic notes (Supplementary Material 2).

### Estimating species richness

In order to predict species richness in the genus, described *Pseudechiniscus* species were categorised into cumulative species richness increasing since the description of *P. suillus* in 1853^32^. Then, a logarithmic best-fit curve was assigned to data to infer a probable progress in the taxonomic descriptions of new taxa. We decided to use this simple method in order to obtain conservative estimates of potential species richness^78^.

### Genotyping

Prior to DNA extraction, all specimens were examined in vivo under PCM. Individual DNA extractions were made from animals and/or exuviae following recent protocols^79,80^. Three nuclear DNA markers were sequenced: 18S rRNA, 28S rRNA and ITS-1. All fragments were amplified using the primers listed in Supplementary Table 3. We attempted to sequence the mitochondrial COI, but obtained good quality chromatograms only for ca. one third of the analysed populations (see Supplementary Table 3 for all used primer combinations). Nevertheless, GenBank accession numbers for obtained COI sequences are presented alongside the nuclear markers in Supplementary Table 4, and in cases of successful COI amplification, the barcode was used to verify species identities in our dataset. Sequencing products were read with the ABI 3130xl sequencer at the Molecular Ecology Lab, Institute of Environmental Sciences of the Jagiellonian University. Sequences were processed in BioEdit ver. 7.2.5^81^.

### Species delimitation

For the purpose of molecular species discrimination, ITS-1-based (710 bp) phylogenetic tree was reconstructed using IQ-TREE^82,83^ under the best-fit GTR + F + G4 model indicated by ModelFinder^84^. A bPTP model was used^85^ with the following priors: tree rooted on several outgroup genera (see below), 100 thousand MCMC generations and 0.1 burn-in. Results of bPTP are presented on the concatenated phylogenetic trees as the clades recovered in both analyses were identical (topology of deeper nodes is irrelevant from the perspective of species delimitation).

### Phylogenetics

The 18S rRNA and 28S rRNA sequences were aligned using the default settings and the G-INS-I method of MAFFT version 7^86,87^ [https://mafft.cbrc.jp/alignment/server/] and manually checked in BioEdit. We were not able to use sequences from earlier studies^20,21^ as they represented non-homologous fragments of both markers (however, all available COI sequences were separately aligned with two outgroup taxa: *Acanthechiniscus islandicus* and *Echiniscus testudo*). ITS-1 sequences were aligned using ClustalW Multiple Alignment tool^88^ implemented in BioEdit. Seven outgroup taxa representing various echiniscid genera were chosen: *Bryodelphax australasiaticus*, *Diploechiniscus oihonnae*, *Echiniscus succineus*, *Echiniscus testudo*, *Hypechiniscus gladiator*, *Mopsechiniscus granulosus*, *Testechiniscus spitsbergensis*. Then, the aligned sequences were trimmed to: 884 bp (18S rRNA), 713 bp (28S rRNA), 710 bp (ITS-1), and subsequently concatenated using SequenceMatrix^89^ (we concatenated sequences exclusively within specimens, i.e. all three concatenated markers always originated from a single animal). Phylogeny of the genus *Pseudechiniscus* was reconstructed using Maximum Likelihood (ML) and Bayesian Inference (BI) methods. IQ-TREE was applied in ML, and Model-Finder indicated the retention of three separate partitions^90^: SYM+I+G4 (18S rRNA), SYM+I+G4 (28S rRNA), GTR+F+G4 (ITS-1); K3Pu+F+I+G4 model was applied in the COI phylogeny presented in Supplementary Material 2. BI was performed both in MrBayes^91^ and BEAST^92^. In MrBayes, random starting trees were used and the analysis was run for ten million generations, sampling the Markov chain every 1000 generations. An average standard deviation of split frequencies of < 0.01 was used as a guide to ensure the two independent analyses had converged. The program Tracer v.1.6^93^ was then used to ensure Markov chains had reached stationarity and to determine the correct “burn-in” for the analysis, which was the first 10% of generations. The effective sample size values were greater than 200, and a consensus tree was obtained after summarising the resulting topologies and discarding the “burn-in”. The original matrix was also analysed using BEAST in order to obtain a set of Bayesian phylogenetic trees needed for biogeographic analyses. Four clock and tree prior combinations were chosen and ran in parallel: (a) random local clock^94^ with the coalescent tree prior, (b) random local clock with the speciation: Yule process as the tree prior, (c) strict clock^95^ with the coalescent tree prior and (d) strict clock with the speciation: Yule process as the tree prior. Tree searches ran for 10 million generations, sampling a tree each 1000 steps. These trees were summarised with the TREEANNOTATOR software (distributed with BEAST) [https://beast.community/treeannotator], removing the first 1000 trees. Eventually, combinations (a) and (b) were chosen for further analyses as Markov chains did not reach stationarity in the latter options. All final consensus trees were viewed and visualised in FigTree v.1.4.3 (http://tree.bio.ed.ac.uk/software/figtree). Character states were coded for populations (Supplementary Material 2) and tested for phylogenetic signal^96^ with a consensus tree in RASP^97,98^ with implemented R packages 'adephylo'^99^ and 'geiger'^100^. Only traits indicated as phylogenetically significant were reconstructed on the simplified trees (single lineage representing each species to ensure that the polytomies at the tip nodes were absent) with BayesTraits^29^ implemented in RASP.

### Historical biogeography

1000 most credible BEAST trees were used in RASP analyses. Areas were coded for species and defined broadly as classical biogeographic realms^62^, with the reservation that Palaearctic was divided into West and East Palaearctic as traditionally separated by the Ural Mountains, Caspian Sea and Zagros Mountains. Both S-DIVA and ancestral area reconstruction in BioGeoBEARS were ran, the latter under the DIVALIKE model^101^ as we rejected the DEC+J model with the highest AICc_wt score since it has been criticised due to theoretical problems^102^.

### Ecological biogeography

We used the ecological niche modelling (ENM) approach to predict the current potential distribution of *P. (M.)* cf. *angelusalas* and *P. (P.)* cf. *ehrenbergi*. The ENM was performed with the use of Maxent algorithm, ver. 3.4.1^103^, with the kuenm package^104^, in R^105^. To eliminate a potential bias of clustered occurrences, the datasets were filtered so that there was only one record per a 5 arc-min cell for each species. Thus, only 15 occurrence records for *P. (M.)* cf. *angelusalas* and 12 records for *P. (P.)* cf. *ehrenbergi* were used in modelling (see Supplementary Material 5 for the full list of records). We used the bioclimatic variables available in WorldClim version 2.1^106^ [http://www.worldclim.com/version2], with a 5-arc-minute resolution, as environmental variables for Maxent modelling. Out of 19 available variables, we excluded those that combined temperature and precipitation (bio8, bio9, bio18 and bio19), because they displayed artificial discontinuities between adjacent grid cells in some areas, which could introduce artefacts to modelling^107^. From the remaining 15 bioclimatic variables, we removed the highly correlated ones (Spearman rank correlation value <|0.85|) and selected those with the highest importance based on jack-knife procedure (regularised training gain) for each species (Supplementary Tables 6–7). Finally, we used six variables in the ENM: bio2 (mean diurnal range), bio4 (temperature seasonality), bio10 (mean temperature of the warmest quarter), bio12 (annual precipitation), bio14 (precipitation of the driest month) and bio15 (precipitation seasonality). Based on these variables and randomly selected 60% of occurrence records, we created 255 candidate models for each species combining 17 values of regularisation multiplier (0.1–1.0 at intervals 0.1, 2–6, 8 and 10) and 15 combinations of four feature classes (linear = l, quadratic = q, product = p, and hinge = h). Then, all candidate models were evaluated based on the partial ROC approach^108^ and predictive power of model based on omission rates^109^, using the test data subset (40% of species records not included in training data). Statistically significant models with omission rates ≤ 10% were selected as the best models. The 10 best models for *P. (M.)* cf. *angelusalas* and 4 for the *P. (P.)* cf. *ehrenbergi* were selected according to these criteria (Supplementary Table 8). For each parameter setting selected as the best, we created 10 bootstrap replicates of models with complemental log–log (cloglog) output format^103^. Models were calibrated and projected using the whole world as a training area and all runs were set with 500 iterations and 10,000 background points. Final maps of potential distribution of both species were created in QGIS^110^ by averaging all best models. The map showing similarities in the distribution of both species is provided in Supplementary Material 9.

## Supplementary Information

Supplementary Information 1.

Supplementary Information 2.

Supplementary Information 3.

Supplementary Information 4.

Supplementary Information 5.

Supplementary Information 6.

Supplementary Information 7.

Supplementary Information 8.

Supplementary Information 9.

## Data Availability

Data generated or analysed during this study are included in the published article and its supplementary information files. All unique sequences are deposited in GenBank (i.e. if several specimens from one population shared an identical haplotype, only one sequence was uploaded).
