# Supplementary material for: Phylogeography and morphological evolution of Pseudechiniscus (Heterotardigrada: Echiniscidae)
Source: Sci Rep. 2021 Apr 7;11:7606. doi: 10.1038/s41598-021-84910-6 (PMC8027217; doi:10.1038/s41598-021-84910-6)
Supplement: Supplementary file 1 — Supplementary Information 1. [file 41598_2021_84910_MOESM1_ESM.pdf]

Phylogeography and morphological evolution of *Pseudechiniscus* (Heterotardigrada: Echiniscidae)

Piotr Gąsiorek*†, Katarzyna Vončina*, Krzysztof Zając & Łukasz Michalczyk†‡

Department of Invertebrate Evolution, Institute of Zoology and Biomedical Research, Faculty of Biology, Jagiellonian University, Gronostajowa 9, 30-387 Kraków, Poland

*Equal contribution.

†Corresponding authors: piotr.lukas.gasiorek@gmail.com, LM@tardigrada.net

‡Senior authorship.

Supplementary Table 1. List of populations used in analyses. Types of analyses: (LCM) imaging and morphometry in PCM/NCM, (SEM) imaging in SEM, (DNA) DNA sequencing. Number in each analysis indicates how many specimens were utilised in a given method.

Species	Sample code	Coordinates altitude	Locality	Sample type	Collector	Analyses		
						LCM	SEM	DNA
Pseudechiniscus (M.) sp. 1	ID.411	1°44'59"S 120°32'16"E 701 m asl	Indonesia, Celebes, Sulawesi Tengah, Saluopa Waterfall	moss from tree bark	Artur Oczkowski & Piotr Gąsiorek	12	2	2
Pseudechiniscus (M.) sp. 2	MY.063	5°31'45"N 118°04'19"E 100 m asl	Malaysia, Sabah, Gomantong Caves	moss from tree bark	Piotr Gąsiorek	4	–	4
Pseudechiniscus (M.) sp. 3	MY.588	3°49'21"N 113°46'10"E 35 m asl	Malaysia, Sarawak, Niah Caves	moss from rooting wood	Artur Oczkowski & Piotr Gąsiorek	3	–	1
Pseudechiniscus (M.) sp. 4	ID.057	8°24'46"S 115°25'23"E 592 m asl	Indonesia, Bali, Karangasem Regency	moss from tree bark	Łukasz Michalczyk & Grzegorz Kwiatkowski	22	–	10
Pseudechiniscus (M.) sp. 4	ID.518	1°51'20"S 120°19'30"E 1 311 m asl	Indonesia, Celebes, Sulawesi Tengah, Lore Lindu, Lembah Valley	moss + lichen from tree bark	Artur Oczkowski & Piotr Gąsiorek	16	–	4
Pseudechiniscus (M.) sp. 4	ID.689	3°42'45"S 128°13'24"E 410 m asl	Indonesia, the Moluccas, Ambon, Gunung Soya	moss from tree bark	Łukasz Krzywański & Piotr Gąsiorek	17	–	6
Pseudechiniscus (M.) sp. 4	ID.691	3°42'45"S 128°13'23"E 410 m asl	Indonesia, the Moluccas, Ambon, Gunung Soya	moss from tree bark	Łukasz Krzywański & Piotr Gąsiorek	16	–	9
Pseudechiniscus (M.) sp. 4	ID.693	3°42'45"S 128°13'20"E 415 m asl	Indonesia, the Moluccas, Ambon, Gunung Soya	moss from tree bark	Łukasz Krzywański & Piotr Gąsiorek	24	–	4
Pseudechiniscus (M.) sp. 4	ID.846	3°10'52"S 129°2'58"E 295 m asl	Indonesia, the Moluccas, Seram, the pass between Triana and Jerili/Sawai	lichen from a palm tree	Łukasz Krzywański & Piotr Gąsiorek	55	20	10
Pseudechiniscus (M.) sp. 4	ID.887	0°40'23"N 127°25'08"E 693 m asl	Indonesia, the Moluccas, Tidore, Gunung Kiematubu	moss + lichen from tree bark	Piotr Gąsiorek	6	–	3
Pseudechiniscus	MY.776	1°43'24"N	Malaysia,	moss from	Artur	8	–	4

Species	Sample code	Coordinates altitude	Locality	Sample type	Collector	Analyses		
						LCM	SEM	DNA
(M.) sp. 4		110°28'24"E 56 m asl	Sarawak, Bako Peninsula	rock and soil	Oczkowski & Piotr Gąsiorek			
Pseudechiniscus (M.) sp. 5	MU.001	ca. 20°22'S 57°29'E 580 m asl	Mauritius, vicinity of Mare aux Vacoas	moss from tree bark	Olena Garmish	85	30	18
Pseudechiniscus (M.) sp. 6	ID.842	3°11'02"S 129°02'44"E 230 m asl	Indonesia, the Moluccas, Seram, the pass between Triana and Jerili/Sawai	moss + lichen from tree bark	Łukasz Krzywański & Piotr Gąsiorek	30	20	4
Pseudechiniscus (M.) sp. 6	ID.954	1°22'59"N 124°50'23"E 649 m asl	Indonesia, Celebes, Sulawesi Utara, Kali Waterfall	moss + lichen from a palm tree	Łukasz Krzywański & Piotr Gąsiorek	12	–	6
Pseudechiniscus (M.) sp. 7	MY.026	6°05'31"N 116°32'53"E 3 500 m asl	Malaysia, Sabah, Gunung Kinabalu	moss from tree trunk	Maciej Barczyk	9	–	2
Pseudechiniscus (M.) sp. 8	MY.090	ca. 4°02'N 114°48'E 50–70 m asl	Malaysia, Sarawak, Gunung Mulu	moss from tree trunk	Piotr Gąsiorek	3	–	4
Pseudechiniscus (P.) sp. 9	AT.050	47°07'38"N 10°20'43"E 1 561 m asl	Austria, Tyrol, the valley of Malfonbach	moss from rock	Łukasz Krzywański	15	10	8
Pseudechiniscus (P.) sp. 9	ES.188	36°45'25"N 5°27'05"W 689 m asl	Spain, Andalucía, Sierra de Grazalema	moss + lichen from tree bark	Witold Morek & Piotr Gąsiorek	7	–	10
Pseudechiniscus (P.) sp. 9	ES.202	36°31'20"N 5°39'10"W 369 m asl	Spain, Andalucía, Parque natural de Los Alcornocales, Mediterranean cork oak forest	lichen from tree bark	Witold Morek & Piotr Gąsiorek	3	–	2
Pseudechiniscus (P.) sp. 9	GB.023	57°18'31"N 2°57'18"W 430 m asl	Scotland, Cabrach, Foot of The Buck	lichen from rock	Brian Blagden	9	–	4
Pseudechiniscus (P.) sp. 9	GB.035	57°18'31"N 2°57'18"W 427 m asl	Scotland, Cabrach, Foot of The Buck	lichen from rock	Brian Blagden	37	10	8
Pseudechiniscus (P.) sp. 9	TN.018	36°43'19"N 8°43'30"E 474 m asl	Tunisia, Jendouba Governorate, Beni M'tir	moss from soil	Jamila Marnissi	19	25	10
Pseudechiniscus (P.) sp. 10	ZA.366	33°19'59"S 26°32'35"E	Republic of South Africa, Eastern Cape,	moss + lichen from	Witold Morek & Bartłomiej	17	–	4

Species	Sample code	Coordinates altitude	Locality	Sample type	Collector	Analyses		
						LCM	SEM	DNA
		696 m asl	vicinity of Grahamstown	tree bark	Surmacz			
Pseudechiniscus (P.) sp. 11	ZA.246	29°45'14"S 29°11'33"E 1 901 m asl	Republic of South Africa, KwaZulu-Natal, Drakensberg	moss from rock partially submerged in water	Witold Morek & Bartłomiej Surmacz	4	–	2
Pseudechiniscus (P.) sp. 12	MG.005	12°30'49"S 49°10'56"E 993 m asl	Madagascar,	moss from tree bark	Wojciech Witaliński	4	–	1
Pseudechiniscus (P.) sp. 13	US.036	36°07'17"N 82°05'37"W 1 239 m asl	USA, Tennessee, Roan Mountain	moss from tree bark	Diane Nelson	19	10	4
Pseudechiniscus (P.) sp. 14	ME.008	42°14'51"N 19°4'47"E 42 m asl	Montenegro, vicinity of Virpazar	moss from rock wall	Aleksandra Rysiewska	29	10	13
Pseudechiniscus (P.) sp. 15	PL.189	49°24'17"N 20°32'52"E 595 m asl	Poland, Homole Ravine	moss from rock	Maciej Barczyk	38	5	4
Pseudechiniscus (P.) sp. 16	AR.439	26°17'25"S 53°46'29"W 754 m asl	Argentina, Misiones, vicinity of Campina de América	moss + lichen from tree trunk	Witold Morek & Bartłomiej Surmacz	4	–	2
Pseudechiniscus (P.) sp. 16	BR.016	25°30'52"S 48°27'46"W 4 m asl	Brazil, Paraná, Ilha da Cotinga, mangrove forest	moss from tree bark	Reinhardt M. Kristensen	123	–	10
Pseudechiniscus (P.) sp. 17	US.037	36°06'50"N 82°05'11"W 1 488 m asl	USA, Tennessee, Roan Mountain	moss from tree bark	Diane Nelson	4	–	1
Pseudechiniscus (P.) sp. 18	IT.120	45°28'55"N 7°22'22"E 1 830 m asl	Italy, Piedmont, Lago di Teleccio, Vallone di Piantonetto	moss from rock	Witold Morek & Piotr Gąsiorek	30	–	6
Pseudechiniscus (M.) cf. *angelusalas*	ID.368	0°57'56"S 119°46'32"E 1 284 m asl	Indonesia, Celebes, Sulawesi Tengah, Marowola Mountains	moss from tree bark and tree root	Artur Oczkowski & Piotr Gąsiorek	11	–	6
Pseudechiniscus (M.) cf. *angelusalas*	ID.417	1°44'57"S 120°32'18"E 698 m asl	Indonesia, Celebes, Sulawesi Tengah, Saluopa Waterfall	moss from tree bark	Artur Oczkowski & Piotr Gąsiorek	19	–	6

Species	Sample code	Coordinates altitude	Locality	Sample type	Collector	Analyses		
						LCM	SEM	DNA
Pseudechiniscus (M.) cf. *angelusalas*	ID.483	1°51'42"S 120°19'08"E 1 243 m asl	Indonesia, Celebes, Sulawesi Tengah, Lore Lindu, Lembah Valley	moss from rock	Artur Oczkowski & Piotr Gąsiorek	12	–	6
Pseudechiniscus (M.) cf. *angelusalas*	ID.485	1°51'37"S 120°19'09"E 1 237 m asl	Indonesia, Celebes, Sulawesi Tengah, Lore Lindu, Lembah Valley	moss from soil and tree bark	Artur Oczkowski & Piotr Gąsiorek	8	–	6
Pseudechiniscus (M.) cf. *angelusalas*	ID.526	1°51'16"S 120°19'31"E 1 326 m asl	Indonesia, Celebes, Sulawesi Tengah, Lore Lindu, Lembah Valley	moss from tree bark	Artur Oczkowski & Piotr Gąsiorek	10	–	10
Pseudechiniscus (M.) cf. *angelusalas*	VN.026	11°53'59"N 108°26'57"E 1 316 m asl	Vietnam, Lâm Đồng, Đà Lạt, Datanla Waterfall	moss from rock	Daniel Stec	2	–	2
Pseudechiniscus (M.) cf. *angelusalas*	ZA.177	29°02'57"S 29°24'18"E 1 415 m asl	Republic of South Africa, KwaZulu-Natal, Drakensberg	moss from rock	Witold Morek & Bartłomiej Surmacz	10	10	4
Pseudechiniscus (M.) cf. *angelusalas*	ZA.178	29°03'07"S 29°24'20"E 1 520 m asl	Republic of South Africa, KwaZulu-Natal, Drakensberg	moss from rock	Witold Morek & Bartłomiej Surmacz	10	–	2
Pseudechiniscus (M.) cf. *angelusalas*	ZA.256	29°45'12"S 29°11'17"E 1 942 m asl	Republic of South Africa, KwaZulu-Natal, Drakensberg	moss from rock	Witold Morek & Bartłomiej Surmacz	10	–	4
Pseudechiniscus (P.) asper	JP.012	40°54'04"N 140°51'58"E 30 m asl	Japan, Honshu, Asamushi Prefecture, Aomori	moss from tree bark	Reinhardt M. Kristensen	5	–	2
Pseudechiniscus (P.) cf. *ehrenbergi*	ID.464	1°52'48"S 120°15'48"E 778 m asl	Indonesia, Celebes, Sulawesi Tengah, Lembah Valley, cacao tree plantation	moss from tree bark	Artur Oczkowski & Piotr Gąsiorek	11	–	9
Pseudechiniscus (P.) cf. *ehrenbergi*	ID.466	1°52'39"S 120°15'41"E 766 m asl	Indonesia, Celebes, Sulawesi Tengah,	moss + lichen from tree bark	Artur Oczkowski & Piotr Gąsiorek	7	–	8

Species	Sample code	Coordinates altitude	Locality	Sample type	Collector	Analyses		
						LCM	SEM	DNA
			Lembah Valley, cacao tree plantation					
Pseudechiniscus (P.) cf. *ehrenbergi*	ID.467	1°52'39"S 120°15'41"E 766 m asl	Indonesia, Celebes, Sulawesi Tengah, Lembah Valley, cacao tree plantation	moss + lichen from tree bark	Artur Oczkowski & Piotr Gąsiorek	29	–	13
Pseudechiniscus (P.) cf. *ehrenbergi*	ID.507	1°51'20"S 120°19'25"E 1 331 m asl	Indonesia, Celebes, Sulawesi Tengah, Lore Lindu, Lembah Valley	moss from tree bark	Artur Oczkowski & Piotr Gąsiorek	20	–	7
Pseudechiniscus (P.) cf. *ehrenbergi*	ID.546	1°50'33"S 120°16'34"E 801 m asl	Indonesia, Celebes, Sulawesi Tengah, Lembah Valley, cacao tree plantation	moss from tree bark	Artur Oczkowski & Piotr Gąsiorek	20	–	5
Pseudechiniscus (P.) cf. *ehrenbergi*	ID.547	1°50'33"S 120°16'34"E 801 m asl	Indonesia, Celebes, Sulawesi Tengah, Lembah Valley, cacao tree plantation	moss + lichen from tree bark	Artur Oczkowski & Piotr Gąsiorek	23	10	12
Pseudechiniscus (P.) cf. *ehrenbergi*	ID.548	1°50'33"S 120°16'34"E 801 m asl	Indonesia, Celebes, Sulawesi Tengah, Lembah Valley, cacao tree plantation	moss + lichen from tree bark	Artur Oczkowski & Piotr Gąsiorek	3	–	8
Pseudechiniscus (P.) cf. *ehrenbergi*	MM.010	20°38'28"N 97°04'14"E 1 333 m asl	Myanmar, Shan, Kakku Pagodas	moss + lichen from tree bark	Katarzyna Vončina	1	–	1
Pseudechiniscus (P.) cf. *ehrenbergi*	VN.042	11°57'04"N 108°26'58"E 1 484 m asl	Vietnam, Lâm Đồng, Đà Lạt, Vườn Hoa	moss from tree bark	Daniel Stec	14	10	8
Pseudechiniscus (P.) cf. *ehrenbergi*	ZA.157	30°31'27"S 29°40'43"E 1 335 m asl	Republic of South Africa, KwaZulu-Natal, vicinity of Kokstad	moss + lichen from tree bark	Witold Morek & Bartłomiej Surmacz	12	–	4
Pseudechiniscus (P.) cf. *ehrenbergi*	ZA.183	29°03'30"S 29°24'12"E 1 693 m asl	Republic of South Africa, KwaZulu-	moss + lichen from rock	Witold Morek & Bartłomiej	16	10	6

Species	Sample code	Coordinates altitude	Locality	Sample type	Collector	Analyses		
						LCM	SEM	DNA
			Natal, Drakensberg		Surmacz			
Pseudechiniscus (P.) cf. *ehrenbergi*	ZA.190	29°03'21"S 29°23'54"E 1 771 m asl	Republic of South Africa, KwaZulu-Natal, Drakensberg	moss from tree bark	Witold Morek & Bartłomiej Surmacz	4	–	4
Pseudechiniscus (P.) cf. *ehrenbergi*	ZA.202	29°03'42"S 29°22'57"E 1 974 m asl	Republic of South Africa, KwaZulu-Natal, Drakensberg	moss + lichen from tree bark	Witold Morek & Bartłomiej Surmacz	61	20	8
Pseudechiniscus (P.) cf. *ehrenbergi*	ZA.246	29°45'14"S 29°11'33"E 1 901 m asl	Republic of South Africa, KwaZulu-Natal, Drakensberg	moss from rock partially submerged in water	Witold Morek & Bartłomiej Surmacz	2	–	1
Pseudechiniscus (M.) quadrilobatus	ID.407	1°44'59"S 120°32'16"E 706 m asl	Indonesia, Celebes, Sulawesi Tengah, Saluopa Waterfall	moss from rock	Artur Oczkowski & Piotr Gąsiorek	14	–	4
Pseudechiniscus (M.) quadrilobatus	ID.474	1°51'54"S 120°19'02"E 1 184 m asl	Indonesia, Celebes, Sulawesi Tengah, Lore Lindu, Lembah Valley	moss from rock wall	Artur Oczkowski & Piotr Gąsiorek	114	50	20
Pseudechiniscus (M.) cf. *saltensis*	AR.251	41°55'47"S 71°33'23"W 385 m asl	Argentina, Río Negro, vicinity of El Bolsón, Andean-Patagonian forest	moss from tree trunk	Witold Morek & Bartłomiej Surmacz	106	30	10
Pseudechiniscus (M.) cf. *saltensis*	AR.266	41°55'48"S 71°33'20"W 405 m asl	Argentina, Río Negro, vicinity of El Bolsón, Andean-Patagonian forest	moss from rock	Witold Morek & Bartłomiej Surmacz	22	–	4
Pseudechiniscus (P.) shintai	JP.012	40°54'04"N 140°51'58"E 30 m asl	Japan, Honshu, Asamushi Prefecture, Aomori	moss from tree bark	Reinhardt M. Kristensen	10	–	3
Pseudechiniscus (P.) suillus	GB.008	58°21'08"N 6°35'14"W 27 m asl	Scotland, Hebrides, Isle of Lewis, Loch nan Muilne (South Uist)	moss + lichen from rock	Brian Blagden	40	–	4
Pseudechiniscus (P.) suillus	GB.028	57°12'39"N	Scotland, Invermoriston,	moss from rock	Brian Blagden	24	–	4

Species	Sample code	Coordinates altitude	Locality	Sample type	Collector	Analyses		
						LCM	SEM	DNA
		4°35'59"W<br>20 m asl	Loch Ness					
Pseudechiniscus (P.) P. suillus	NO.002	58°46'54"N<br>6°19'23"E<br>210 m asl	Norway, Byrkjedal	moss from rock	Andrzej Kaźmierski	338	50	10
Pseudechiniscus (P.) suillus	NO.190	68°22'50"N<br>17°12'20"E<br>27 m asl	Norway, Otofjord	moss from rock	Daniel Stec & Witold Morek	26	20	4
