# Supplementary material for: Phylogeography and morphological evolution of Pseudechiniscus (Heterotardigrada: Echiniscidae)
Source: Sci Rep. 2021 Apr 7;11:7606. doi: 10.1038/s41598-021-84910-6 (PMC8027217; doi:10.1038/s41598-021-84910-6)
Supplement: Supplementary file 2 — Supplementary Information 2. [file 41598_2021_84910_MOESM2_ESM.pdf]

Phylogeography and morphological evolution of *Pseudechiniscus* (Heterotardigrada: Echiniscidae)

Piotr Gąsiorek*†, Katarzyna Vončina*, Krzysztof Zając & Łukasz Michalczyk†‡

Department of Invertebrate Evolution, Institute of Zoology and Biomedical Research, Faculty of Biology, Jagiellonian University, Gronostajowa 9, 30-387 Kraków, Poland

*Equal contribution.

†Corresponding authors: piotr.lukas.gasiorek@gmail.com, LM@tardigrada.net

‡Senior authorship.

Supplementary Material 2. Morphological and biogeographic characteristics of species used in analyses. Taxonomic notes on affinities of some taxa are provided below the table (ps IV' = pseudosegmental plate IV').

**Species**	**Sample code**	**Cephalic papilla**	***Striae***	**Posterior margin of the ps IV'**	**Ventral ornamentation pattern**	**Claw spurs**	**Males in population**	**Region**
Pseudechiniscus (M.) sp. 1	ID.411	elongated	present, mostly reduced	with two evident teeth	complicated, with additional epicuticular thickenings	absent	not found	Oriental
Pseudechiniscus (M.) sp. 2	MY.063	elongated	present	smooth	complicated	present, reduced	not found	Oriental
Pseudechiniscus (M.) sp. 3	MY.588	elongated	present	smooth	complicated	present	not found	Oriental
Pseudechiniscus (M.) sp. 4	ID.057	elongated	present	smooth	complicated	present	not found	Oriental
Pseudechiniscus (M.) sp. 4	ID.518	elongated	present	smooth	complicated	present	not found	Oriental
Pseudechiniscus (M.) sp. 4	ID.689	elongated	present	smooth	complicated	present	present	Oriental
Pseudechiniscus (M.) sp. 4	ID.691	elongated	present	smooth	complicated	present	not found	Oriental
Pseudechiniscus (M.) sp. 4	ID.693	elongated	present	smooth	complicated	present	not found	Oriental
Pseudechiniscus (M.) sp. 4	ID.846	elongated	present	smooth	complicated	present	present	Oriental
Pseudechiniscus (M.) sp. 4	ID.887	elongated	present	smooth	complicated	present	not found	Oriental
Pseudechiniscus (M.) sp. 4	MY.776	elongated	present	smooth	complicated	present	present	Oriental
Pseudechiniscus (M.) sp. 5	MU.001	elongated	present	smooth or with two poorly elevated teeth	complicated	present	present	Afrotropical
Pseudechiniscus (M.) sp. 6	ID.842	elongated	present, reduced	usually with two poorly elevated teeth, rarely smooth	complicated	present	not found	Oriental

Species	Sample code	Cephalic papilla	*Striae*	Posterior margin of the ps IV'	Ventral ornamentation pattern	Claw spurs	Males in population	Region
Pseudechiniscus (M.) sp. 6	ID.954	elongated	present, reduced	usually with two poorly elevated teeth, rarely smooth	complicated	present	not found	Oriental
Pseudechiniscus (M.) sp. 7	MY.026	elongated	present, reduced	smooth	complicated	present	present	Oriental
Pseudechiniscus (M.) sp. 8	MY.090	elongated	present, reduced	smooth	complicated	present	present	Oriental
Pseudechiniscus (P.) sp. 9	AT.050	pseudohemispherical	absent	smooth	reduced	present	present	Palaearctic
Pseudechiniscus (P.) sp. 9	ES.188	pseudohemispherical	absent	smooth	reduced	present	present	Palaearctic
Pseudechiniscus (P.) sp. 9	ES.202	pseudohemispherical	absent	smooth	reduced	present	present	Palaearctic
Pseudechiniscus (P.) sp. 9	GB.023	pseudohemispherical	extremely reduced, discernible only in the caudal plate	smooth, secondarily thickened or with a longitudinal lobe	reduced	present	present	Palaearctic
Pseudechiniscus (P.) sp. 9	GB.035	pseudohemispherical	absent	smooth	reduced	present	present	Palaearctic
Pseudechiniscus (P.) sp. 9	TN.018	pseudohemispherical	absent	smooth or secondarily thickened	reduced	present	present	Palaearctic
Pseudechiniscus (P.) sp. 10	ZA.366	pseudohemispherical	absent	with single longitudinal lobe	complicated	present	present	Afrotropical
Pseudechiniscus (P.) sp. 11	ZA.246	pseudohemispherical	absent	smooth	complicated	present	not found	Afrotropical
Pseudechiniscus (P.) sp. 12	MG.005	pseudohemispherical	absent	smooth	complicated	present, reduced	present	Afrotropical
Pseudechiniscus (P.) sp. 13	US.036	pseudohemispherical	absent	smooth	complicated	present	present	Nearctic
Pseudechiniscus (P.) sp. 14	ME.008	pseudohemispherical	absent	smooth	complicated	present	present	Palaearctic

Species	Sample code	Cephalic papilla	*Striae*	Posterior margin of the ps IV’	Ventral ornamentation pattern	Claw spurs	Males in population	Region
Pseudechiniscus (P.) sp. 15	PL.189	pseudohemispherical	absent	smooth	complicated	present	present	Palaearctic
Pseudechiniscus (P.) sp. 16	AR.439	pseudohemispherical	absent	smooth	complicated	present	not found	Neotropical
Pseudechiniscus (P.) sp. 16	BR.016	pseudohemispherical	extremely reduced, discernible only in the caudal plate	smooth	complicated	present	present	Neotropical
Pseudechiniscus (P.) sp. 17	US.037	pseudohemispherical	absent	with two evident teeth	complicated	present	not found	Nearctic
Pseudechiniscus (P.) sp. 18	IT.120	pseudohemispherical	absent	smooth	complicated	present	present	Palaearctic
Pseudechiniscus (M.) cf. *angelusalas*	ID.368	elongated	present	smooth	complicated	present	not found	Oriental
Pseudechiniscus (M.) cf. *angelusalas*	ID.417	elongated	present	smooth	complicated	present	not found	Oriental
Pseudechiniscus (M.) cf. *angelusalas*	ID.483	elongated	present	smooth	complicated	present	not found	Oriental
Pseudechiniscus (M.) cf. *angelusalas*	ID.485	elongated	present	smooth	complicated	present	not found	Oriental
Pseudechiniscus (M.) cf. *angelusalas*	ID.526	elongated	present	smooth	complicated	present	not found	Oriental
Pseudechiniscus (M.) cf. *angelusalas*	VN.026	elongated	present	smooth or secondarily thickened	complicated	present	not found	Oriental
Pseudechiniscus (M.) cf. *angelusalas*	ZA.177	elongated	present	smooth or secondarily thickened	complicated	present	present	Afrotropical
Pseudechiniscus (M.) cf. *angelusalas*	ZA.178	elongated	present	smooth or secondarily thickened	complicated	present	present	Afrotropical
Pseudechiniscus (M.) cf. *angelusalas*	ZA.256	elongated	present	smooth or secondarily thickened	complicated	present	present	Afrotropical

Species	Sample code	Cephalic papilla	*Striae*	Posterior margin of the ps IV’	Ventral ornamentation pattern	Claw spurs	Males in population	Region
Pseudechiniscus (P.) asper	JP.012	pseudohemispherical	absent	with two evident teeth	complicated	present, reduced	present	Palaearctic
Pseudechiniscus (P.) cf. *ehrenbergi*	ID.464	pseudohemispherical	absent	smooth	complicated	present	not found	Oriental
Pseudechiniscus (P.) cf. *ehrenbergi*	ID.466	pseudohemispherical	absent	smooth	complicated	present	not found	Oriental
Pseudechiniscus (P.) cf. *ehrenbergi*	ID.467	pseudohemispherical	absent	smooth	complicated	present	present	Oriental
Pseudechiniscus (P.) cf. *ehrenbergi*	ID.507	pseudohemispherical	absent	smooth	reduced	present	present	Oriental
Pseudechiniscus (P.) cf. *ehrenbergi*	ID.546	pseudohemispherical	absent	smooth	reduced	present	not found	Oriental
Pseudechiniscus (P.) cf. *ehrenbergi*	ID.547	pseudohemispherical	absent	smooth	reduced	present	present	Oriental
Pseudechiniscus (P.) cf. *ehrenbergi*	ID.548	pseudohemispherical	absent	smooth	reduced	present	not found	Oriental
Pseudechiniscus (P.) cf. *ehrenbergi*	MM.010	pseudohemispherical	absent	smooth	complicated	present	present	Oriental
Pseudechiniscus (P.) cf. *ehrenbergi*	VN.042	pseudohemispherical	absent	smooth or secondarily thickened	complicated	present	not found	Oriental
Pseudechiniscus (P.) cf. *ehrenbergi*	ZA.157	pseudohemispherical	absent	smooth	reduced	present	not found	Afrotropical
Pseudechiniscus (P.) cf. *ehrenbergi*	ZA.183	pseudohemispherical	absent	smooth	reduced	present	not found	Afrotropical
Pseudechiniscus (P.) cf. *ehrenbergi*	ZA.190	pseudohemispherical	absent	smooth	reduced	present	not found	Afrotropical
Pseudechiniscus (P.) cf. *ehrenbergi*	ZA.202	pseudohemispherical	absent	smooth	reduced	present	not found	Afrotropical
Pseudechiniscus (P.) cf. *ehrenbergi*	ZA.246	pseudohemispherical	absent	smooth	reduced	present	not found	Afrotropical
Pseudechiniscus (M.) quadrilobatus	ID.407	elongated, swollen	present	with large lobe	absent	absent	not found	Oriental

Species	Sample code	Cephalic papilla	*Striae*	Posterior margin of the ps IV’	Ventral ornamentation pattern	Claw spurs	Males in population	Region
Pseudechiniscus (M.) quadrilobatus	ID.474	elongated, swollen	present	with large lobe	absent	absent	not found	Oriental
Pseudechiniscus (M.) cf. *saltensis*	AR.251	elongated	present	with two evident teeth	complicated	present	present	Neotropical
Pseudechiniscus (M.) cf. *saltensis*	AR.266	elongated	present	with two evident teeth	complicated	present	present	Neotropical
Pseudechiniscus (P.) shintai	JP.012	pseudohemispherical	absent	smooth	complicated	present	present	Palaearctic
Pseudechiniscus (P.) suillus	GB.008	pseudohemispherical	absent	secondarily thickened	reduced, uniform pillars covering entire venter	present	present	Palaearctic
Pseudechiniscus (P.) suillus	GB.028	pseudohemispherical	absent	secondarily thickened	reduced, uniform pillars covering entire venter	present	present	Palaearctic
Pseudechiniscus (P.) suillus	NO.002	pseudohemispherical	absent	smooth	complicated	present	present	Palaearctic
Pseudechiniscus (P.) suillus	NO.190	pseudohemispherical	absent	smooth	complicated	present	present	Palaearctic

1. After the examination of the type material of *P. (M.) quadrilobatus*[1], *P. gullii*[2] and the description of *P. pilatoi*[3], combined with obtaining new populations from Celebes (the Malay Archipelago), there are no morphological arguments that would substantiate keeping these three species as separate entities. Specifically: (i) the sculpturing is homogeneous in all three species, consisting of widely spaced endocuticular pillars joined by evident *striae*; (ii) the posterior margin of the scapular plate exhibits considerable intra-population variability that exceeds inter-specific variation (the posterior margin may form a lobe, a dimple, or be completely smooth); and (iii), claws have an identical morphology, being very delicate and short, spurless, which is unusual for Echiniscidae, and for *Pseudechiniscus* in particular (= a homoplasious autapomorphy). To conclude, in our opinion, *P. gullii* and *P. pilatoi* are younger synonyms of *P. (M.) quadrilobatus* that is characterised by a wide tropical distribution in the Indopacific area.

2. *Pseudechiniscus (M.)* sp. 5 bears much resemblance to *Pseudechiniscus (M.) santomensis*[4] from the Gulf of Guinea. However, males are unknown for the latter, and the posterior margin of pseudosegmental plate IV’ is smooth in females of *Pseudechiniscus (M.)* sp. 5, whereas the plate is equipped with two teeth in *Pseudechiniscus (M.) santomensis*.

3. *Pseudechiniscus (M.)* sp. 6 is similar to *Pseudechiniscus (M.) bidenticulatus*[5] described from Java, however the *Pseudechiniscus (M.)* sp. 6 has evident spurs on internal claws of whereas the claws in *Pseudechiniscus (M.) bidenticulatus* are spurless, thus species are not conspecific.

4. *Pseudechiniscus (P.)* sp. 17 is very characteristic because of lateral bumps in the positions *B–E*, which, together with the place of origin (Roan Mountain, Tennessee, USA), suggest it is conspecific with an undescribed *Pseudechiniscus* species mentioned in the description of *Pseudechiniscus (P.) brevimontanus*[6].

5. Results of COI comparisons (see the phylogeny below):

– *Pseudechiniscus (P.)* cf. *ehrenbergi* likely corresponds with *Pseudechiniscus (P.) ehrenbergi* s.s. (the morphological disparities embrace e.g. different ventral sculpturing patterns)[7];

– *Pseudechiniscus (P.)* sp. 18 likely corresponds with *Pseudechiniscus* aff. *suillus* (Ca1)[8], especially that both taxa were found in Italy.

6. All remaining unnamed species were identified as new.

References

[1]Iharos, G. Einige Angaben zur Tardigradenfauna Vietnams. *Opusc. Zool.* **9**, 273–277 (1969).

[2]Pilato, G. & Lisi, O. Notes on some tardigrades from southern Mexico with description of three new species. *Zootaxa* **1236**, 53–68 (2006).

[3]Li, X. Tardigrades from the Tsinling Mountains, central China with descriptions of two new species of Echiniscidae (Tardigrada). *J. Nat. Hist*. **41**, 2719–2739 (2007).

[4]Fontoura, P., Pilato, G. & Lisi, O. First record of Tardigrada from São Tomé (Gulf of Guinea, Western Equatorial Africa) and description of *Pseudechiniscus santomensis* sp. nov. (Heterotardigrada: Echiniscidae). *Zootaxa* **2564**, 31–42 (2010).

[5]Bartoš, E. Die Tardigraden der Chinesischen und Javanischen Moosproben. *Acta Soc. Zool. Bohem*. **27**, 108–114 (1963).

[6]Kendall-Fite, K. & Nelson, D.R. Two new species of tardigrades from Short Mountain, Tennessee, U.S.A. *Zool. J. Linn. Soc*. **116**, 205–214 (1996).

[7]Roszkowska, M. *et al*. Integrative description of five *Pseudechiniscus* species (Heterotardigrada: Echiniscidae: the *suillus-facettalis* complex). *Zootaxa* **4763**, 451–484 (2020).

[8]Cesari, M. *et al*. An integrated study of the biodiversity within the *Pseudechiniscus suillus–facettalis* group (Heterotardigrada: Echiniscidae). *Zool. J. Linn. Soc*. **188**, 717–732 (2020).

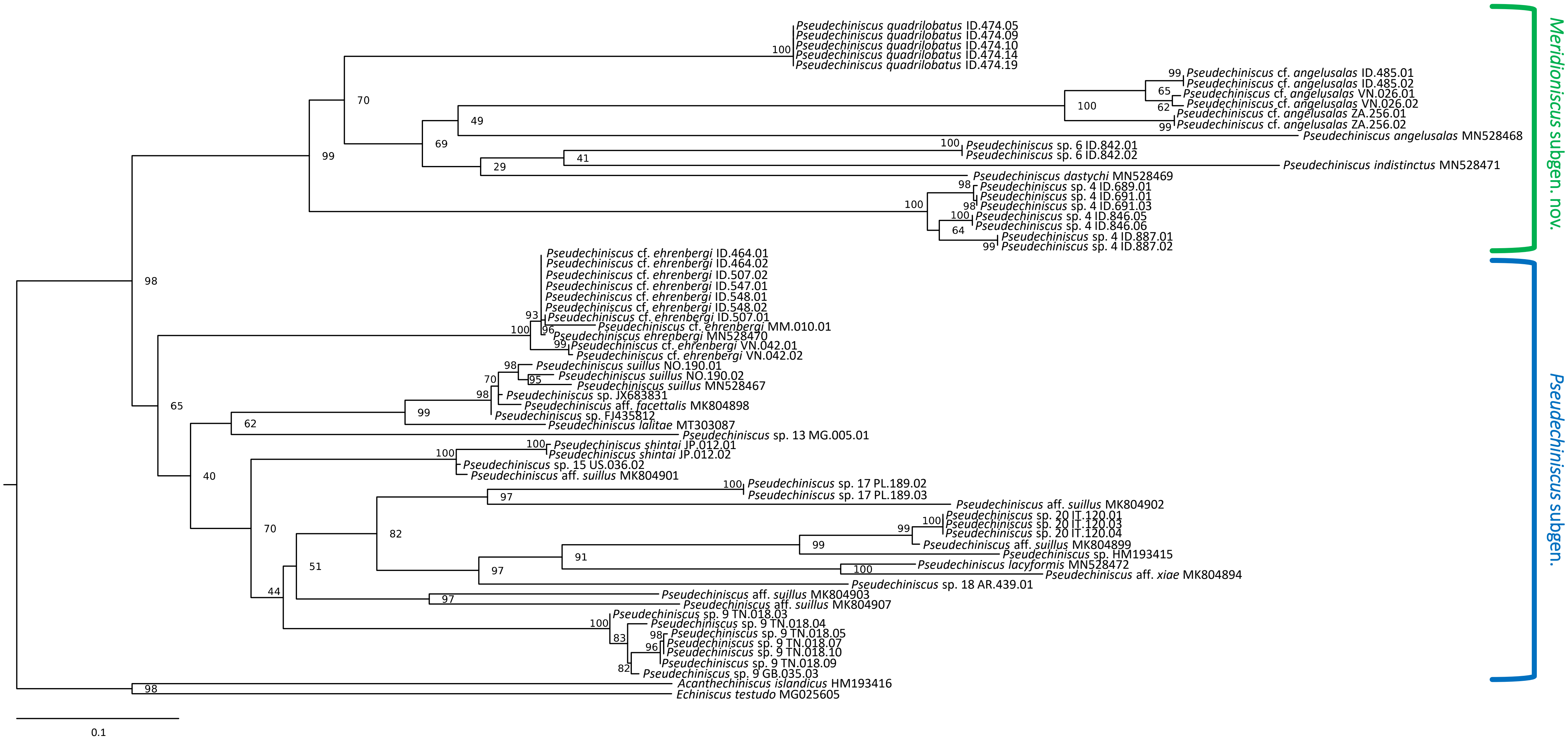

Pseudechiniscus quadrilobatus ID.474.05
Pseudechiniscus quadrilobatus ID.474.09
Pseudechiniscus quadrilobatus ID.474.10
Pseudechiniscus quadrilobatus ID.474.14
Pseudechiniscus quadrilobatus ID.474.19
Pseudechiniscus cf. angelusalas ID.485.01
Pseudechiniscus cf. angelusalas ID.485.02
Pseudechiniscus cf. angelusalas VN.026.01
Pseudechiniscus cf. angelusalas VN.026.02
Pseudechiniscus cf. angelusalas ZA.256.01
Pseudechiniscus cf. angelusalas ZA.256.02
Pseudechiniscus angelusalas MN528468
Pseudechiniscus sp. 6 ID.842.01
Pseudechiniscus sp. 6 ID.842.02
Pseudechiniscus indistinctus MN528471
Pseudechiniscus dastychi MN528469
Pseudechiniscus sp. 4 ID.689.01
Pseudechiniscus sp. 4 ID.691.01
Pseudechiniscus sp. 4 ID.691.03
Pseudechiniscus sp. 4 ID.846.05
Pseudechiniscus sp. 4 ID.846.06
Pseudechiniscus sp. 4 ID.887.01
Pseudechiniscus sp. 4 ID.887.02
Meridioniscus subgen. nov.
Pseudechiniscus cf. ehrenbergi ID.464.01
Pseudechiniscus cf. ehrenbergi ID.464.02
Pseudechiniscus cf. ehrenbergi ID.507.02
Pseudechiniscus cf. ehrenbergi ID.547.01
Pseudechiniscus cf. ehrenbergi ID.548.01
Pseudechiniscus cf. ehrenbergi ID.548.02
Pseudechiniscus cf. ehrenbergi ID.507.01
Pseudechiniscus cf. ehrenbergi MM.010.01
Pseudechiniscus ehrenbergi MN528470
Pseudechiniscus cf. ehrenbergi VN.042.01
Pseudechiniscus cf. ehrenbergi VN.042.02
Pseudechiniscus suillus NO.190.01
Pseudechiniscus suillus NO.190.02
Pseudechiniscus suillus MN528467
Pseudechiniscus sp. JX683831
Pseudechiniscus aff. facettalis MK804898
Pseudechiniscus sp. FJ435812
Pseudechiniscus lalitae MT303087
Pseudechiniscus sp. 13 MG.005.01
Pseudechiniscus shintai JP.012.01
Pseudechiniscus shintai JP.012.02
Pseudechiniscus sp. 15 US.036.02
Pseudechiniscus aff. suillus MK804901
Pseudechiniscus sp. 17 PL.189.02
Pseudechiniscus sp. 17 PL.189.03
Pseudechiniscus aff. suillus MK804902
Pseudechiniscus sp. 20 IT.120.01
Pseudechiniscus sp. 20 IT.120.03
Pseudechiniscus sp. 20 IT.120.04
Pseudechiniscus aff. suillus MK804899
Pseudechiniscus sp. HM193415
Pseudechiniscus lacyformis MN528472
Pseudechiniscus aff. xiae MK804894
Pseudechiniscus sp. 18 AR.439.01
Pseudechiniscus aff. suillus MK804903
Pseudechiniscus aff. suillus MK804907
Pseudechiniscus sp. 9 TN.018.03
Pseudechiniscus sp. 9 TN.018.04
Pseudechiniscus sp. 9 TN.018.05
Pseudechiniscus sp. 9 TN.018.07
Pseudechiniscus sp. 9 TN.018.10
Pseudechiniscus sp. 9 TN.018.09
Pseudechiniscus sp. 9 GB.035.03
Pseudechiniscus subgen.
Acanthechiniscus islandicus HM193416
Echiniscus testudo MG025605
0.1
