# Supplementary material for: Phylogeography and morphological evolution of Pseudechiniscus (Heterotardigrada: Echiniscidae)
Source: Sci Rep. 2021 Apr 7;11:7606. doi: 10.1038/s41598-021-84910-6 (PMC8027217; doi:10.1038/s41598-021-84910-6)
Supplement: Supplementary file 3 — Supplementary Information 3. [file 41598_2021_84910_MOESM3_ESM.pdf]

Phylogeography and morphological evolution of *Pseudechiniscus* (Heterotardigrada: Echiniscidae)

Piotr Gąsiorek*[†], Katarzyna Vončina*, Krzysztof Zając & Łukasz Michalczyk[†‡]

Department of Invertebrate Evolution, Institute of Zoology and Biomedical Research, Faculty of Biology, Jagiellonian University, Gronostajowa 9, 30-387 Kraków, Poland

*Equal contribution.

[†]Corresponding authors: piotr.lukas.gasiorek@gmail.com, LM@tardigrada.net

[‡]Senior authorship.

Supplementary Table 3. Primers and references for specific protocols for amplification of the four DNA fragments sequenced in the study.

DNA fragment	Primer name	Primer direction	Primer sequence (5'-3')	Primer source	PCR programme*
**18S rRNA**	18S_Tar_Ff1	forward	AGGCGAAACCGCGAATGGCTC	Stec et al. (2017)	Zeller (2010)
	18S_Tar_Rr2	reverse	CTGATCGCCTTCGAACCTCTAACTTTCG	Gąsiorek et al. (2017)	
**28S rRNA**	28S_Eutar_F	forward	ACCCGCTGAACTTAAGCATAT	Gąsiorek et al. (2018)	Mironov et al. (2012)
	28SR0990	reverse	CCTTGGTCCGTGTTTCAAGAC	Mironov et al. (2012)	
**ITS-1**	ITS1_Echi_F	forward	CCGTCGCTACTACCGATTGG	Gąsiorek et al. (2019)	Wełnicz et al. (2011)
	ITS1_Echi_R	reverse	GTTCAGAAAACCCTGCAATTCACG		
**COI**	bcdF01	forward	CATTTTCHACTAAYCATAARGATATTGG	Dabert et al. (2008)	Wełnicz et al. (2011)
	bcdR04	reverse	TATAAACYTCDGGATGNCCAAAAAA		
	LCO1490_JJ	forward	CHACWAAYCATAAAGATATYGG	Astrin & Stüben (2008)	Wełnicz et al. (2011)
	HCO2198_JJ	reverse	AWACTTCVGGRTGVCCAAARAATCA		
	LCO 1490	forward	GGTCAACAAATCATAAAGATATTGG	Folmer et al. (1994)	Grobys et al. (2020)
	HCO 2198	reverse	TAAACTTCAGGGTGACCAAAAAATCA		
	LCO 1490	forward	GGTCAACAAATCATAAAGATATTGG	Folmer et al. (1994)	Cesari et al. (2020)
	HCOout	reverse	CCAGGTAAAATTAAAATATAAACTTC	Carpenter & Wheeler (1999)	

* – All PCR programmes are also provided in Stec et al. (2020).

Underlined primer pairs did not amplify material in the case of COI.

Astrin J.J. & Stüben P. (2008) Phylogeny in cryptic weevils: molecules, morphology and new genera of Western Palaearctic Cryptorhynchinae (Coleoptera: Curculionidae). Invertebrate Systematics 22: 503–522. http://doi.org/10.1071/is07057

Carpenter J.M. & Wheeler W.C. (1999) Towards simultaneous analysis of molecular and morphological data in Hymenoptera. Zoologica Scripta 28: 251–260. https://doi.org/10.1046/j.1463-6409.1999.00009.x

Cesari M., Montanari M., Kristensen R.M., Bertolani R., Guidetti R. & Rebecchi L. (2020) An integrated study of the biodiversity within the *Pseudechiniscus suillus–facettalis* group (Heterotardigrada: Echiniscidae). Zoological Journal of the Linnean Society 188: 717–732. https://doi.org/10.1093/zoolinnean/zlz045

Dabert J., Ehrnsberger R. & Dabert M. (2008) *Glaucalges tytonis* sp. nov. (Analgoidea: Xolalgidae) from the barn owl *Tyto alba* (Strigiformes: Tytonidae): compiling morphology with DNA barcode data for taxa descriptions in mites (Acari). Zootaxa 1719: 41–52. https://doi.org/10.11646/zootaxa.1719.1.2

Folmer O., Black M., Hoeh W., Lutz R. & Vrijenhoek R. (1994) DNA primers for amplification of mitochondrial cytochrome c oxidase subunit I from diverse metazoan invertebrates. Molecular Marine Biology and Biotechnology 3: 294–9.

Gąsiorek P., Jackson K.J., Meyer H.A., Zając K., Nelson D.R., Kristensen R.M. & Michalczyk Ł. (2019) *Echiniscus virginicus* complex: the first case of pseudocryptic allopatry and pantropical distribution in tardigrades. Biological Journal of the Linnean Society 128: 789–805. https://doi.org/10.1093/biolinnean/blz147

Gąsiorek P., Stec D., Morek W. & Michalczyk Ł. (2017) An integrative redescription of *Echiniscus testudo* (Doyère, 1840), the nominal taxon for the class Heterotardigrada (Ecdysozoa: Panarthropoda: Tardigrada). Zoologischer Anzeiger 270: 107–122. https://doi.org/10.1016/j.jcz.2017.09.006

Gąsiorek P., Stec D., Zawierucha K., Kristensen R.M. & Michalczyk Ł. (2018) Revision of *Testechiniscus* Kristensen, 1987 (Tardigrada: Heterotardigrada: Echiniscidae) refutes the polar–temperate distribution of the genus. Zootaxa 4472: 261–297. https://doi.org/10.11646/zootaxa.4472.2.3

Grobys D., Roszkowska M., Gawlak M., Kmita H., Kepel A., Kepel M., Parnikoza I., Bartylak T. & Kaczmarek Ł. (2020) High diversity in the *Pseudechiniscus suillus–facettalis* complex (Heterotardigrada: Echiniscidae) with remarks on the morphology of the genus *Pseudechiniscus*. Zoological Journal of the Linnean Society 188: 733–752. https://doi.org/10.1093/zoolinnean/zlz171

Mironov S.V., Dabert J. & Dabert M. (2012) A new feather mite species of the genus *Proctophyllodes* Robin, 1877 (Astigmata: Proctophyllodidae) from the long-tailed tit *Aegithalos caudatus* (Passeriformes: Aegithalidae): morphological description with DNA barcode data. Zootaxa 3253: 54–61. https://doi.org/10.11646/zootaxa.3253.1.2

Stec D., Zawierucha K. & Michalczyk Ł. (2017) An integrative description of *Ramazzottius subanomalus* (Biserov, 1985) (Tardigrada) from Poland. Zootaxa 4300: 403–420. https://doi.org/10.11646/zootaxa.4300.3.4

Wełnicz W., Grohme M.A., Kaczmarek Ł., Schill R.O. & Frohme M. (2011) ITS-2 and18S rRNA data from *Macrobiotus polonicus* and *Milnesium tardigradum* (Eutardigrada, Tardigrada). Journal of Zoological Systematics and Evolutionary Research 49: 34–39. https://doi.org/10.1111/j.1439-0469.2010.00595.x

Zeller C. (2010) Untersuchung der Phylogenie von Tardigraden anhand der Genabschnitte 18S rDNA und Cytochrom c Oxidase Untereinheit 1 (COX I). MScThesis, Technische Hochschule Wildau, Wildau.
