# Supplementary material for: Phylogeography and morphological evolution of Pseudechiniscus (Heterotardigrada: Echiniscidae)
Source: Sci Rep. 2021 Apr 7;11:7606. doi: 10.1038/s41598-021-84910-6 (PMC8027217; doi:10.1038/s41598-021-84910-6)
Supplement: Supplementary file 4 — Supplementary Information 4. [file 41598_2021_84910_MOESM4_ESM.pdf]

Phylogeography and morphological evolution of *Pseudechiniscus* (Heterotardigrada: Echiniscidae)

Piotr Gąsiorek*[†], Katarzyna Vončina*, Krzysztof Zając & Łukasz Michalczyk[†‡]

Department of Invertebrate Evolution, Institute of Zoology and Biomedical Research, Faculty of Biology, Jagiellonian University, Gronostajowa 9, 30-387 Kraków, Poland

*Equal contribution.

[†]Corresponding authors: piotr.lukas.gasiorek@gmail.com, LM@tardigrada.net

[‡]Senior authorship.

Supplementary Table 4. GenBank accession numbers for DNA markers. If there is a single haplotype deposited for a given species per population, it means that no haplotype variability was detected (all sequences uploaded to GenBank originate from single specimens).

Sample code	Species	18S rRNA	28S rRNA	ITS-1	COI
AR.251	*Pseudechiniscus (M.)* cf. *saltensis*	MW031920	MW032009	MW032099	–
AR.266	*Pseudechiniscus (M.)* cf. *saltensis*	MW031921	MW032010	MW032100	–
AR.439	*Pseudechiniscus (P.)* sp. 16	MW031922	MW032011	MW032101	MW031172
AT.050	*Pseudechiniscus (P.)* sp. 9	MW031923–5	MW032012–4	MW032102–4	–
BR.016	*Pseudechiniscus (P.)* sp. 16	MW031926	MW032015	MW032105	–
ES.188	*Pseudechiniscus (P.)* sp. 9	MW031927–8	MW032016–7	MW032106–7	–
ES.202	*Pseudechiniscus (P.)* sp. 9	MW031929	MW032018	MW032108	–
GB.008	*Pseudechiniscus (P.) suillus*	MW031930–1	MW032019–20	MW032109–10	–
GB.023	*Pseudechiniscus (P.)* sp. 9	MW031932	MW032021	MW032111	–
GB.028	*Pseudechiniscus (P.) suillus*	MW031933–4	MW032022–3	MW032112–3	–
GB.035	*Pseudechiniscus (P.)* sp. 9	MW031935–6	MW032024–5	MW032114–5	MW031173
ID.057	*Pseudechiniscus (M.)* sp. 4	MW031937	MW032026	MW032116	–
ID.368	*Pseudechiniscus (M.)* cf. *angelusalas*	MW031938	MW032027	MW032117	–
ID.407	*Pseudechiniscus (M.) quadrilobatus*	MW031939	MW032028	MW032118	–
ID.411	*Pseudechiniscus (M.)* sp. 1	MW031940	MW032029	MW032119	–
ID.417	*Pseudechiniscus (M.)* cf. *angelusalas*	MW031941	MW032030	MW032120	–
ID.464	*Pseudechiniscus (P.)* cf. *ehrenbergi*	MW031942	MW032031	MW032121	MW031174–5
ID.467	*Pseudechiniscus (P.)* cf. *ehrenbergi*	MW031943	MW032032	MW032122	–
ID.474	*Pseudechiniscus (M.) quadrilobatus*	MW031944–6	MW032033–5	MW032123–5	MW031176–80
ID.483	*Pseudechiniscus (M.)* cf. *angelusalas*	MW031947	MW032036	MW032126	–
ID.485	*Pseudechiniscus (M.)* cf. *angelusalas*	MW031948	MW032037	MW032127	MW031181–2
ID.507	*Pseudechiniscus (P.)* cf. *ehrenbergi*	MW031949	MW032038	MW032128	MW031183–4
ID.518	*Pseudechiniscus (M.)* sp. 4	MW031950	MW032039	MW032129	–
ID.526	*Pseudechiniscus (M.)* cf. *angelusalas*	MW031951	MW032040	MW032130	–
ID.546	*Pseudechiniscus (P.)* cf. *ehrenbergi*	MW031952	MW032041	MW032131	–
ID.547	*Pseudechiniscus (P.)* cf. *ehrenbergi*	MW031953–5	MW032042–4	MW032132–4	MW031185
ID.548	*Pseudechiniscus (P.)* cf. *ehrenbergi*	MW031956	MW032045	MW032135	MW031186–7
ID.689	*Pseudechiniscus (M.)* sp. 4	MW031957	MW032046	MW032136	MW031188
ID.691	*Pseudechiniscus (M.)* sp. 4	MW031958	MW032047	MW032137	MW031189–90

Sample code	Species	18S rRNA	28S rRNA	ITS-1	COI
ID.693	*Pseudechiniscus (M.)* sp. 4	MW031959	MW032048	MW032138	–
ID.842	*Pseudechiniscus (M.)* sp. 6	MW031960	MW032049	MW032139	MW031191–2
ID.846	*Pseudechiniscus (M.)* sp. 4	MW031961	MW032050	MW032140	MW031193–4
ID.887	*Pseudechiniscus (M.)* sp. 4	MW031962	MW032051	MW032141	MW031195–6
ID.954	*Pseudechiniscus (M.)* sp. 6	MW031963	MW032052	MW032142	–
IT.120	*Pseudechiniscus (P.)* sp. 18	MW031964	MW032053	MW032143	MW031197–9
JP.012	*Pseudechiniscus (P.) asper*	MT645083	MT645081	MT645085	–
	Pseudechiniscus (P.) shintai	MT645084	MT645082	MT645086	MT644270–1
ME.008	*Pseudechiniscus (P.)* sp. 14	MW031965–9	MW032054–8	MW032144–8	–
MG.005	*Pseudechiniscus (P.)* sp. 12	MW031970	MW032059	MW032149	MW031200
MM.010	*Pseudechiniscus (P.)* cf. *ehrenbergi*	MW031971	MW032060	MW032150	MW031201
MU.001	*Pseudechiniscus (M.)* sp. 5	MW031972	MW032061	MW032151	–
MY.026	*Pseudechiniscus (M.)* sp. 7	MW031973	MW032062	MW032152	–
MY.063	*Pseudechiniscus (M.)* sp. 2	MW031974	MW032063	MW032153	–
MY.090	*Pseudechiniscus (M.)* sp. 8	MW031975	MW032064	MW032154	–
MY.588	*Pseudechiniscus (M.)* sp. 3	MW031976	MW032065	MW032155	–
MY.776	*Pseudechiniscus (M.)* sp. 4	MW031977	MW032066	MW032156	–
NO.002	*Pseudechiniscus (P.) suillus*	MW031978–81	MW032067–70	MW032157–60	–
NO.190	*Pseudechiniscus (P.) suillus*	MW031982–5	MW032071–4	MW032161–4	MW031202–3
PL.189	*Pseudechiniscus (P.)* sp. 15	MW031986–7	MW032075–6	MW032165–6	MW031204–5
TN.018	*Pseudechiniscus (P.)* sp. 9	MW031988–9	MW032077–8	MW032167–8	MW031206–11
US.036	*Pseudechiniscus (P.)* sp. 13	MW031990	MW032079	MW032169	MW031212
US.037	*Pseudechiniscus (P.)* sp. 17	MW031991	MW032080	MW032170	–
VN.026	*Pseudechiniscus (M.)* cf. *angelusalas*	MW031992–3	MW032081–2	MW032171–2	MW031213–4
VN.042	*Pseudechiniscus (P.)* cf. *ehrenbergi*	MW031994–6	MW032083–5	MW032173–5	MW031215–6
ZA.157	*Pseudechiniscus (P.)* cf. *ehrenbergi*	MW031997	MW032086	MW032176	–
ZA.177	*Pseudechiniscus (M.)* cf. *angelusalas*	MW031998–9	MW032087–8	MW032177–8	–
ZA.178	*Pseudechiniscus (M.)* cf. *angelusalas*	MW032000	MW032089	MW032179	–
ZA.183	*Pseudechiniscus (P.)* cf. *ehrenbergi*	MW032001	MW032090	MW032180	–
ZA.190	*Pseudechiniscus (P.)* cf. *ehrenbergi*	MW032002	MW032091	MW032181	–
ZA.202	*Pseudechiniscus (P.)* cf. *ehrenbergi*	MW032003–4	MW032092–3	MW032182–3	–
ZA.246	*Pseudechiniscus (P.)* sp. 11	–	MW032094	MW032184	–
	Pseudechiniscus (P.) cf. *ehrenbergi*	MW032005	MW032095	MW032185	–
ZA.256	*Pseudechiniscus (M.)* cf.	MW032006	MW032096	MW032186	MW031217–8

Sample code	Species	18S rRNA	28S rRNA	ITS-1	COI
	angelusalas				
ZA.366	*Pseudechiniscus (P.)* sp. 10	MW032007–8	MW032097–8	MW032187–8	–
