# Supplementary material for: Phylogeography and morphological evolution of Pseudechiniscus (Heterotardigrada: Echiniscidae)
Source: Sci Rep. 2021 Apr 7;11:7606. doi: 10.1038/s41598-021-84910-6 (PMC8027217; doi:10.1038/s41598-021-84910-6)
Supplement: Supplementary file 6 — Supplementary Information 6. [file 41598_2021_84910_MOESM6_ESM.pdf]

Phylogeography and morphological evolution of *Pseudechiniscus* (Heterotardigrada: Echiniscidae)

Piotr Gąsiorek*[†], Katarzyna Vončina*, Krzysztof Zając & Łukasz Michalczyk[†‡]

Department of Invertebrate Evolution, Institute of Zoology and Biomedical Research, Faculty of Biology, Jagiellonian University, Gronostajowa 9, 30-387 Kraków, Poland

*Equal contribution.

[†]Corresponding authors: piotr.lukas.gasiorek@gmail.com, LM@tardigrada.net

[‡]Senior authorship.

Supplementary Table 6. Spearman correlation coefficients for bioclimatic variables selected to use in ecological niche modelling for *Pseudechiniscus (M.)* cf. *angelusalas* and *Pseudechiniscus (P.)* cf. *ehrenbergi*; coefficients > |0.85| are marked in red font and bioclimatic variables selected for model calibration and projection are bolded.

Variable	bio 1	**bio 2**	bio 3	**bio 4**	bio 5	bio 6	bio 7	**bio 10**	bio 11	**bio 12**	bio 13	**bio 14**	**bio 15**	bio 16	bio 17
bio 1		0.599	0.768	-0.622	0.959	0.980	-0.445	0.975	0.986	0.610	0.600	0.393	-0.230	0.636	0.406
**bio 2**	**0.599**		0.591	**-0.177**	0.703	0.493	0.095	**0.652**	0.533	**0.180**	0.246	**0.015**	**0.092**	0.256	-0.003
bio 3	0.768	0.591		-0.857	0.670	0.803	-0.701	0.682	0.809	0.481	0.524	0.133	0.020	0.518	0.192
**bio 4**	-0.622	**-0.177**	-0.857		-0.456	-0.718	0.952	**-0.491**	-0.706	**-0.442**	-0.453	**-0.090**	**0.007**	-0.445	-0.174
bio 5	0.959	0.703	0.670	-0.456		0.899	-0.249	0.993	0.913	0.494	0.483	0.318	-0.208	0.525	0.322
bio 6	0.980	0.493	0.803	-0.718	0.899		-0.571	0.923	0.998	0.663	0.639	0.436	-0.274	0.674	0.462
bio 7	-0.445	0.095	-0.701	0.952	-0.249	-0.571		-0.299	-0.547	-0.396	-0.398	-0.073	0.000	-0.383	-0.161
**bio 10**	0.975	**0.652**	0.682	**-0.491**	0.993	0.923	-0.299		0.934	**0.525**	0.514	**0.345**	**-0.218**	0.554	0.350
bio 11	0.986	0.533	0.809	-0.706	0.913	0.998	-0.547	0.934		0.659	0.641	0.421	-0.255	0.675	0.445
**bio 12**	0.610	**0.180**	0.481	**-0.442**	0.494	0.663	-0.396	**0.525**	0.659		0.958	**0.769**	**-0.440**	0.978	0.818
bio 13	0.600	0.246	0.524	-0.453	0.483	0.639	-0.398	0.514	0.641	0.958		0.629	-0.220	0.990	0.676
**bio 14**	0.393	**0.015**	0.133	**-0.090**	0.318	0.436	-0.073	**0.345**	0.421	**0.769**	0.629		**-0.768**	0.676	0.968
**bio 15**	**-0.230**	**0.092**	0.020	**0.007**	-0.208	-0.274	0.000	**-0.218**	-0.255	**-0.440**	-0.220	**-0.768**		-0.292	-0.774
bio 16	0.636	0.256	0.518	-0.445	0.525	0.674	-0.383	0.554	0.675	0.978	0.990	0.676	-0.292		0.719
bio 17	0.406	-0.003	0.192	-0.174	0.322	0.462	-0.161	0.350	0.445	0.818	0.676	0.968	-0.774	0.719	

Supplementary Table 7. Results of the jackknife test of variable importance according to regularized training gain with each variable used in isolation for *Pseudechiniscus (M.)* cf. *angelusalas* and *Pseudechiniscus (P.)* cf. *ehrenbergi*. Variables selected for model calibration and projection are bolded.

Variable	Regularised training gain	
	P. (M.) cf. *angelusalas*	*P. (P.)* cf. *ehrenbergi*
bio1	1.503	1.443
**bio2**	**1.086**	**1.094**
bio3	1.927	1.712
**bio4**	**2.041**	**1.922**
bio5	1.493	1.372
bio6	1.723	1.642
bio7	1.984	1.780
**bio10**	**1.836**	**1.832**
bio11	1.815	1.788
**bio12**	**1.254**	**1.166**
bio13	1.147	1.090
**bio14**	**1.120**	**1.071**
**bio15**	**1.147**	**1.107**
bio16	1.163	1.107
bio17	1.116	1.071

Supplementary Table 8. Results of selection of the best settings of the regularization multiplier and feature classes in the Maxent for modelling of *Pseudechiniscus (M.)* cf. *angelusalas* and *Pseudechiniscus (P.)* cf. *ehrenbergi*, according to such criteria as statistical significance and omission rate at 10%.

Model	Mean AUC ratio	Partial ROC	Omission rate	Number of parameters
Pseudechiniscus (M.) cf. *angelusalas*				
M_0.1_F_h	1.994	0	0	42
M_0.1_F_lh	1.994	0	0	45
M_0.1_F_qh	1.994	0	0	39
M_0.1_F_ph	1.994	0	0	56
M_0.1_F_lqh	1.994	0	0	47
M_0.1_F_lph	1.994	0	0	52
M_0.1_F_qph	1.994	0	0	53
M_0.1_F_lqph	1.994	0	0	47
M_0.2_F_h	1.992	0	0	24
M_0.2_F_lh	1.992	0	0	20
Pseudechiniscus (P.) cf. *ehrenbergi*				
M_0.7_F_h	1.984	0	0	60
M_0.8_F_h	1.983	0	0	58
M_0.9_F_h	1.983	0	0	57
M_1_F_h	1.985	0	0	61
