# Supplementary material for: Phylogeography and morphological evolution of Pseudechiniscus (Heterotardigrada: Echiniscidae)
Source: Sci Rep. 2021 Apr 7;11:7606. doi: 10.1038/s41598-021-84910-6 (PMC8027217; doi:10.1038/s41598-021-84910-6)
Supplement: Supplementary file 7 — Supplementary Information 7. [file 41598_2021_84910_MOESM7_ESM.pdf]

Phylogeography and morphological evolution of *Pseudechiniscus* (Heterotardigrada: Echiniscidae)

Piotr Gąsiorek*†, Katarzyna Vončina*, Krzysztof Zając & Łukasz Michalczyk†‡

Department of Invertebrate Evolution, Institute of Zoology and Biomedical Research, Faculty of Biology, Jagiellonian University, Gronostajowa 9, 30-387 Kraków, Poland

*Equal contribution.

†Corresponding authors: piotr.lukas.gasiorek@gmail.com, LM@tardigrada.net

‡Senior authorship.

Supplementary Material 9. Potential distribution area of *Pseudechiniscus (M.)* cf. *angelusalas* and *Pseudechiniscus (P.)* cf. *ehrenbergi* predicted according to averaged value of equal training sensitivity and specificity threshold (0.2645 for *Pseudechiniscus (M.)* cf. *angelusalas* and 0.7670 for *Pseudechiniscus (P.)* cf. *ehrenbergi*). Generated using Maxent, ver. 3.4.1[103] [https://biodiversityinformatics.amnh.org/open_source/maxent/].
